# Non-monotonic Response to Monotonic Stimulus: Regulation of Glyoxylate Shunt Gene-Expression Dynamics in *Mycobacterium tuberculosis*

**DOI:** 10.1371/journal.pcbi.1004741

**Published:** 2016-02-22

**Authors:** Joao A. Ascensao, Pratik Datta, Baris Hancioglu, Eduardo Sontag, Maria L. Gennaro, Oleg A. Igoshin

**Affiliations:** 1 Department of Bioengineering and Center for Theoretical Biological Physics, Rice University, Houston, Texas, United States of America; 2 Public Health Research Institute, New Jersey Medical School, Rutgers University, Newark, New Jersey, United States of America; 3 Department of Mathematics and Center for Quantitative Biology, Rutgers University, Piscataway, New Jersey, United States of America; University of Illinois at Urbana-Champaign, UNITED STATES

## Abstract

Understanding how dynamical responses of biological networks are constrained by underlying network topology is one of the fundamental goals of systems biology. Here we employ monotone systems theory to formulate a theorem stating necessary conditions for non-monotonic time-response of a biochemical network to a monotonic stimulus. We apply this theorem to analyze the non-monotonic dynamics of the σ^B^-regulated glyoxylate shunt gene expression in *Mycobacterium tuberculosis* cells exposed to hypoxia. We first demonstrate that the known network structure is inconsistent with observed dynamics. To resolve this inconsistency we employ the formulated theorem, modeling simulations and optimization along with follow-up dynamic experimental measurements. We show a requirement for post-translational modulation of σ^B^ activity in order to reconcile the network dynamics with its topology. The results of this analysis make testable experimental predictions and demonstrate wider applicability of the developed methodology to a wide class of biological systems.

## Author Summary

Over the last several years mathematical modeling has become widely used to understand how biochemical systems respond to perturbations. In particular, dynamics of the response, i.e. the precise nature of how the responses changes with time, has become the focus of multiple studies. However, to this date only a few general rules that relate the dynamical responses with the structure of the underlying networks have been formulated. To this end, we ask which properties of the network allow systems to have a non-monotonic time-response (first increasing and then decreasing) to a monotonically increasing signal. We show that the networks displaying such responses must include indirect negative feedback or incoherent feedforward loop. Applying this result to the measured non-monotonic expression for glyoxylate shunt genes in *Mycobacterium tuberculosis*, a network known to be important to mycobacterial virulence, we show that the currently postulated network structure does not match the predictions of the theorem. Using a combination of mathematical modeling and follow-up experimental test we predict a novel incoherent loop in the network. This methodology has wide applications outside the specific network studied in this work—the theorem may potentially simplify the analysis of many biological systems.

## Introduction

Uncovering how regulatory networks shape the dynamical properties of cellular responses to external stimuli is one of the ultimate goals of system biology. Despite our successes in mapping and modeling gene regulatory networks for a wide variety of model systems, only a handful of generalizable rules relating network architecture to its dynamic performance have been formulated. These rules are often called evolutionary design principles of biochemical networks [1]. Developing new approaches to find these design principles should allow us to extend our understanding of biological network dynamics from a few case studies to a wide variety of model systems. Here we formulate one such approach and apply it to a network controlling the response of *Mycobacterium tuberculosis*, the causative agent of tuberculosis (TB), to hypoxic stress.

With almost one-third of the world population infected, TB remains a major public health threat [2]. *M*. *tuberculosis* survives stress conditions induced by host immunity by undergoing major metabolic and physiological remodeling that leads to mycobacterial dormancy [3–6]. Understanding this adaptive response of the tubercle bacillus is central to our long-term ability to control the pathogen. Transcriptional networks downstream of the alternative sigma factor σ^E^, are critical for this adaptive response. They are activated when bacteria infect host macrophages, and induce the production of virulence factors and host inflammatory responses [7,8]. Deletion of *sigE* leads to the strongest attenuation of *M*. *tuberculosis* murine infection among all accessory sigma factor mutant strains [7]. Induction of σ^E^ can be studied *in vitro* by exposing *M*. *tuberculosis* cells to a wide range of stressors such as hypoxia and surface or oxidative stress [7,8].

Due to its importance for cellular survival, σ^E^ is subjected to rather complex regulatory mechanisms at both the transcriptional and post-translational levels. Transcription of *sigE* is controlled by three different promoters (P1-3). P1 is responsible for basal expression of *sigE* under normal physiological conditions. P2 is activated in the presence of MprA, part of the MprAB two component system that can sense surface stress [9,10]. Interestingly, σ^E^ also activates the transcription of MprAB, forming a positive feedback loop. The last promoter (P3) is transcribed by the σ^H^-RNAP holoenzyme in response to oxidative stress. σ^E^ post-translational regulation is primarily controlled by its anti-sigma factor, RseA. RseA binds to and sequesters σ^E^, preventing the formation of the active σ^E^-RNAP holoenzyme. However, under stressful conditions, PknB (a eukaryotic-like Ser/Thr protein kinase) will phosphorylate RseA, tagging it for degradation by the ClpC1P2 proteases [10,11].

**Fig 1:**
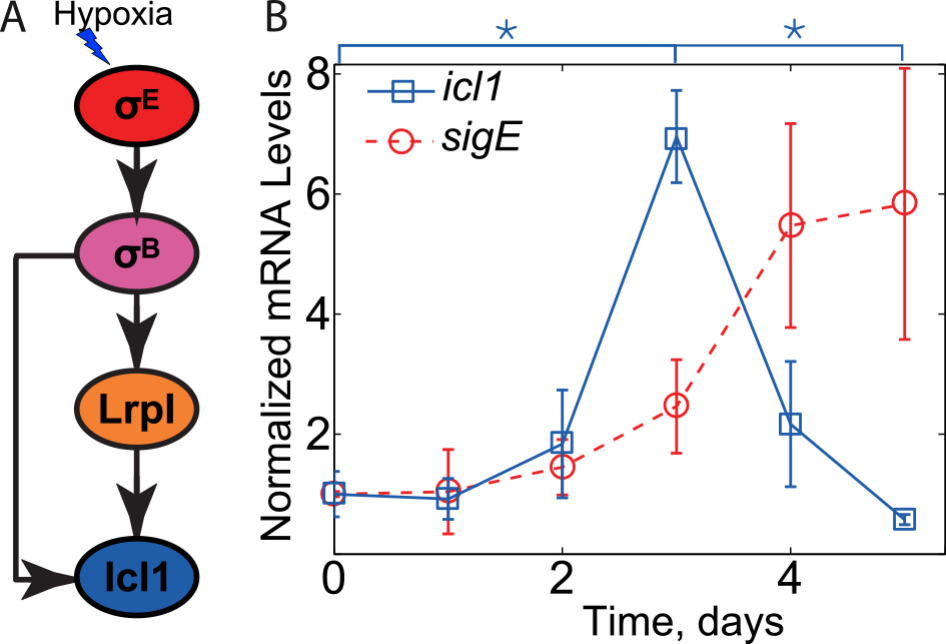
Non-monotonic induction of *icl1*. (A) Structure of *icl1* transcriptional regulation network. The central metabolism gene *icl1* was previously thought to be solely controlled by a simple feedforward network under hypoxic conditions. Arrows represent positive transcriptional regulation. (B) Expression measurements for *icl1* and the accessory sigma factor *sigE* mRNA (data from [12]). Three independent experiments were performed, and the means (error bars are ± standard deviations) are reported, normalized to the mean value of the first data point. While *sigE* copy number is increasing, *icl1* peaks at day 3 and decreases after that. (*p≤0.05).

Recently, the transcriptional dynamics of σ^E^ and several of its regulon members following hypoxic stress have been quantified, and the networks controlling production of two critical central metabolism genes, *icl1* (Rv0467, glyoxylate shunt) and *gltA1* (Rv1131, methylcitrate cycle), have been characterized [12]. These genes are implicated in the growth-phase-dependent metabolic adaptation of *M*. *tuberculosis* [13] and are essential for growth and persistence of tubercle bacilli in infection models [14–16]. The glyoxylate shunt is especially important because it allows *M*. *tuberculosis* to efficiently metabolize fatty acids; indeed, it has been suggested that fatty acids may be the major source of carbon and energy for tubercle bacilli in chronically infected lung tissue [16]. For *icl1* (Fig 1A), transcription requires both σ^B^, an alternative sigma factor transcribed under σ^E^ control, and a σ^B^-regulated transcription factor, named *lrpI* (Rv0465c, local regulatory protein of *icl1*) [12]. This network motif–coherent feedforward loop–is a common motif of bacterial regulatory networks that is known to produce delays in responses to step-up inputs, to filter transient stimuli, and to increase network sensitivity [1,17]. Notably, in the case of *icl1*, the resulting activation dynamics is different from that is seen for other coherent feedforward loops. Following gradual depletion of oxygen over the course of 3 days, which leads to σ^E^ activation, *icl1* is transiently induced on day 4, and then decreases to pre-induction levels by day 5 (Fig 1B). A similar transient surge in *icl1* has also been observed *in vivo* [12].

In this paper, we develop a methodology to uncover the mechanism of non-monotonic response following the monotonic dynamics of a stimulus, and apply it to the transient surge in *icl1* dynamics. We first formulate a general theorem based on monotone systems theory that gives a necessary condition for this observation. Then we employ a combination of modeling, parameter optimization, and experimental tests to uncover missing interactions in the *icl1* regulatory network and to make new experimentally testable predictions.

## Results

### Monotone systems theory predicts inconsistency between known network structure and observed dynamics

A major challenge in the quantitative analysis of biological systems is their tendency to be highly complex and non-linear, complicating analysis of system behavior. Therefore, there is a substantial need for methods that can make it possible to constrain potential network topologies based on the observed dynamics without knowledge of the underlying parameters. Here we develop a generalized theorem that gives a necessary condition for non-monotonic system dynamics, given a monotonic input signal. In this section, we semi-intuitively describe the concept and main results. A comprehensive formulation and the proof of the theorem are found in the S1 Text.

A function is defined as monotonic if it is increasing or decreasing over a given domain. That is, for a monotonically increasing function, for all *t*_1_ ≥ *t*_0_, *f*(*t*_1_) ≥ *f*(*t*_0_). For monotonically decreasing functions, the sign of the inequality is flipped, i.e. *f*(*t*_1_) ≤ *f*(*t*_0_). If the function is defined by a dynamical system, in the sense that it is a component of a solution of a set of ordinary differential equations, we set to identify general properties of the dynamical system that ensure monotonic increase/decrease of its components over time. In the context of biological networks, the components usually represent concentrations of biological species (e.g. mRNA, proteins, metabolites, etc.). The dynamical system consists of biochemical kinetics equations for these species:

$$\frac{d}{dt}X_{1}\left(t\right)=F_{1}\left(X_{1}\left(t\right),X_{2}\left(t\right),\ldots ,X_{n}\left(t\right);u\left(t\right)\right)$$ (1)

$$\begin{array}{c} \frac{d}{dt}X_{2}\left(t\right)=F_{2}\left(X_{1}\left(t\right),X_{2}\left(t\right),\ldots ,X_{n}\left(t\right);u\left(t\right)\right)\\ \vdots \\ \frac{d}{dt}X_{n}\left(t\right)=F_{n}\left(X_{1}\left(t\right),X_{2}\left(t\right),\ldots ,X_{n}\left(t\right);u\left(t\right)\right) \end{array}$$

Here *X*_*i*_(*t*) are time-dependent concentrations of relevant chemical species and *F*_*i*_ is the net flux into a given concentration pool, i.e. net sum of all the rates of reactions producing specie *i* minus all the rates of reactions that consume it. The function *u*(*t*) is a known time-dependent input into this biochemical system, such as an externally supplied chemical ligand or stressor. Usually, it will only directly affect one or a few network nodes. For generality we allow multiple nodes to be directly affected by *u*(*t*); these would be called the 'input variables’. On the other hand, the last node *X*_*n*_(*t*) is arbitrarily designated as the 'output variable’. For brevity, we can represent this dynamical system in the vector-notation as

$$\frac{d}{dt}X(t)=F(X(t);u(t))$$ (2)

where **X** and **F** are vectors with components *X*_*i*_ and *F*_*i*_ respectively.

**Fig 2:**
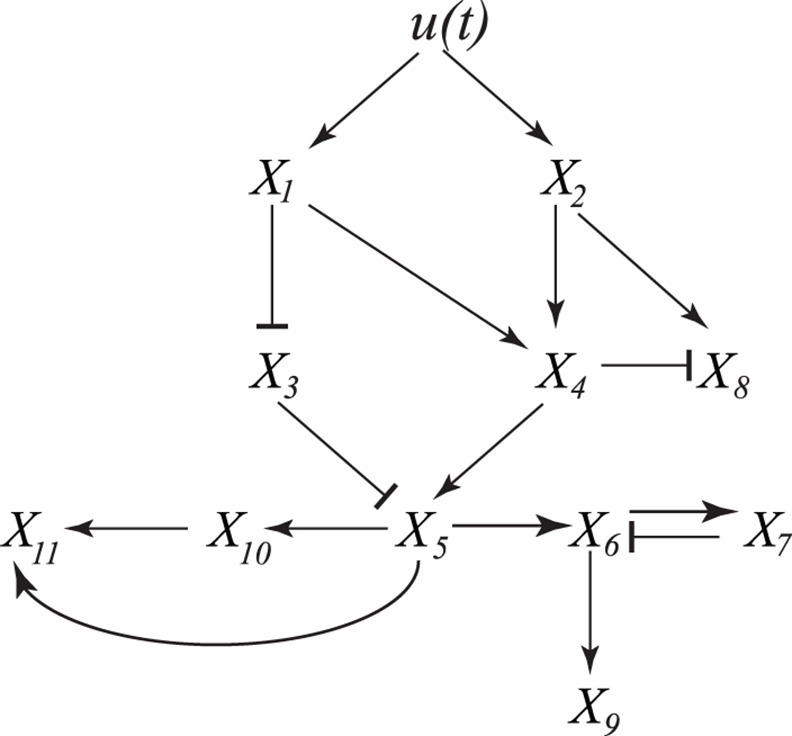
Sample graphical representation of a dynamical system to illustrate the theorem. The input node *u(t)* is a known input function. The dynamics of the dependent variables *X*_*i*_(*t*) are given by equations of the form of Eq 1. Arrows connect the nodes connect nodes with non-zero partial derivatives; pointed and blunt arrows correspond to positive and negative partial derivatives respectively (see text for details).

We may represent this dynamical system as a graph consisting of (n+1) nodes and directed edges connecting some of the nodes (such as the network diagram on Fig 1A). The nodes correspond to the input function *u*(*t*) and all chemical species *X*_*i*_(*t*) (*i = 1…n*). The edges correspond to biochemical interactions: the input node corresponding to *u*(*t*) is connected to all input variables, i.e. all variables for which ∂*F*_*i*_/∂*u* ≠ 0. Analogously, the node corresponding to concentration *X*_*k*_ will be connected by a directed edge from *X*_*l*_ if ∂*F*_*k*_/∂*X*_*l*_ ≠ 0. If the increase in the concentration of species *l* increases the production flux for concentration of *k* (i.e., ∂*F*_*k*_/∂*X*_*l*_ ≥ 0), then we draw an edge with an arrow (↓) or assigned parity +1. Biologically, this corresponds to “activation”. Conversely, if *j* inhibits/represses species *i* (i.e., ∂*F*_*i*_/∂*X*_*j*_ ≤ 0), then we will draw an edge with blunt arrow (┴) or assigned parity −1. Biologically, this corresponds to repression. Similarly, the parity can be assigned to the edges from *u*(*t*) to input variables based on the sign of ∂*F*_*i*_/∂*u*. For simplicity, we restrict our attention to systems in which the signs of partial derivatives ∂*F*_*i*_/∂*u* and ∂*F*_*i*_/∂*X*_*j*_ are the same over the entire domain in which functions *X*_*j*_(*t*) and *u*(*t*) take their values. Note that self-loops, i.e. edges that connect nodes to itself, are not included in such representation; thus the sign of ∂*F*_*i*_/∂*X*_*i*_ is irrelevant. Fig 2 illustrates such graph for a particular system.

Directed paths, i.e. sets of consecutive edges, of such graph describe how signal *u*(*t*) propagates through the dynamical system. If all the directed paths to the output variable node *X*_*n*_(*t*) go through a node *X*_*i*_(*t*), then perturbations to these node that make it insensitive to the signal (e.g. gene knockout that sets *X*_*i*_(*t*)) would also imply that *X*_*n*_(*t*) is insensitive to the signal. For example, in Fig 2 all directed paths from signal to nodes *X*_6_,*X*_7_,*X*_9_,*X*_10_ and *X*_11_ go through *X*_5_. For each directed path in the graph consisting of multiple edges we can define a combined parity corresponding to a product of individual parities of edges from which each path consists. For example, on Fig 2 the directed paths from *X*_1_ to *X*_4_ to *X*_5_ and from *X*_1_ to *X*_3_ to *X*_5_ are positive whereas the directed path from *X*_2_ to *X*_4_ to *X*_8_ is negative. Note that directed paths can include full circles around indirect feedback loops, e.g. from *X*_5_ to *X*_6_ to *X*_7_ to *X*_6_ to *X*_9_.

With these definitions and notation in mind we are ready to formulate a general theorem that states a sufficient condition for the system output *X*_*n*_(*t*) to be a monotonic function of time. For convenience, two alternative (but mathematically equivalent) formulations of the theorem are given–one for the case in which the input signal *u(t)* is known and another one for which internal node *X*_*i*_(*t*) serves as a proxy for some unknown signal.

**Theorem.** For a dynamical system given by Eq (2) which is initially in steady state (i.e. **F**(**X**(0);*u*(0)) = **0**) the response of the output *X*_*n*_(*t*) will monotonically increase (or decrease) in time response to changes in the input *u*(*t*) if:

(1) The input function *u*(*t*) is monotonically increasing in time and all the directed paths from input node *u*(*t*) to the output node *X*_*n*_(*t*) have the same parity. Furthermore, monotonically increasing *u(t)* will trigger monotonic increase of *X*_*n*_(*t*) if parity is positive or will trigger monotonic decrease if parity is negative.

or

(2) All the directed paths from the input nodes to the output node pass through an internal node *X*_*i*_(*t*) with monotonically increasing or decreasing dynamics and all the directed paths from *X*_*i*_(*t*) to the output node *X*_*n*_(*t*) have the same parity. Furthermore, monotonically increasing (*X*_*i*_(*t*) will result in monotonic increase of *X*_*n*_(*t*) if parity is positive or will result in monotonic decrease if parity is negative. On the other hand, monotonically decreasing (*X*_*i*_(*t*) will result in monotonic decrease of *X*_*n*_(*t*) if parity is positive or in monotonic increase if parity is negative.

We note that if *u(t)* is a monotonically decreasing function simple change of variables (e.g. *u→ −u)* will result in monotonically increasing input and the theorem can still be used.

For example, on Fig 2 the first formulation for the theorem allow us to conclude that monotonically increasing input *u*(*t*) will ensure monotonic increase of, for instance, *X*_4_(*t*), since both directed paths from *u* to *X*_4_ (via *X*_1_ or *X*_2_) have positive parity. By repeating this reasoning for the remaining nodes, we can conclude that monotonic increase is ensured for *X*_1_,*X*_2_,*X*_4_,*X*_5_,*X*_10_ and *X*_11_, monotonic decrease is ensured for *X*_3_, but monotonicity cannot be guaranteed for *X*_6_,*X*_7_,*X*_8_ and *X*_9_. On the other hand, if we do not know whether input signal *u*(*t*) is monotonic or in case an additional negative path in the network from *u*(*t*) to *X*_5_ is added, we may still use the second formulation to conclude that if *X*_5_(*t*) is monotonic so will be *X*_10_ and *X*_11_. Indeed, all the paths to *X*_10_ and *X*_11_ from input *u*(*t*) pass through *X*_5_ and all the paths from *X*_5_ to *X*_10_ and *X*_11_ have positive parity. The argument does not work for *X*_9_ due to a negative feedback loop between *X*_6_ and *X*_7_ (a directed path that goes around this loop will have the opposite parity from the path that does not).

One straightforward consequence of this theorem states that for *any* dynamical system in which a certain output variable *X*_*n*_(*t*) behaves non-monotonically as a function of time in response to a monotonic signal, there must be at least two (undirected) paths between that node and input node *u*(*t*) with different parities, i.e. one with an odd number and another with an even number (or zero) of negative interactions. Such paths can only exist if the corresponding graph exhibits incoherent feedforward loops and/or negative feedback loops. This property can be very useful to identify that known biochemical network diagram is inconsistent with measured dynamics as we illustrate below.

**Fig 3:**
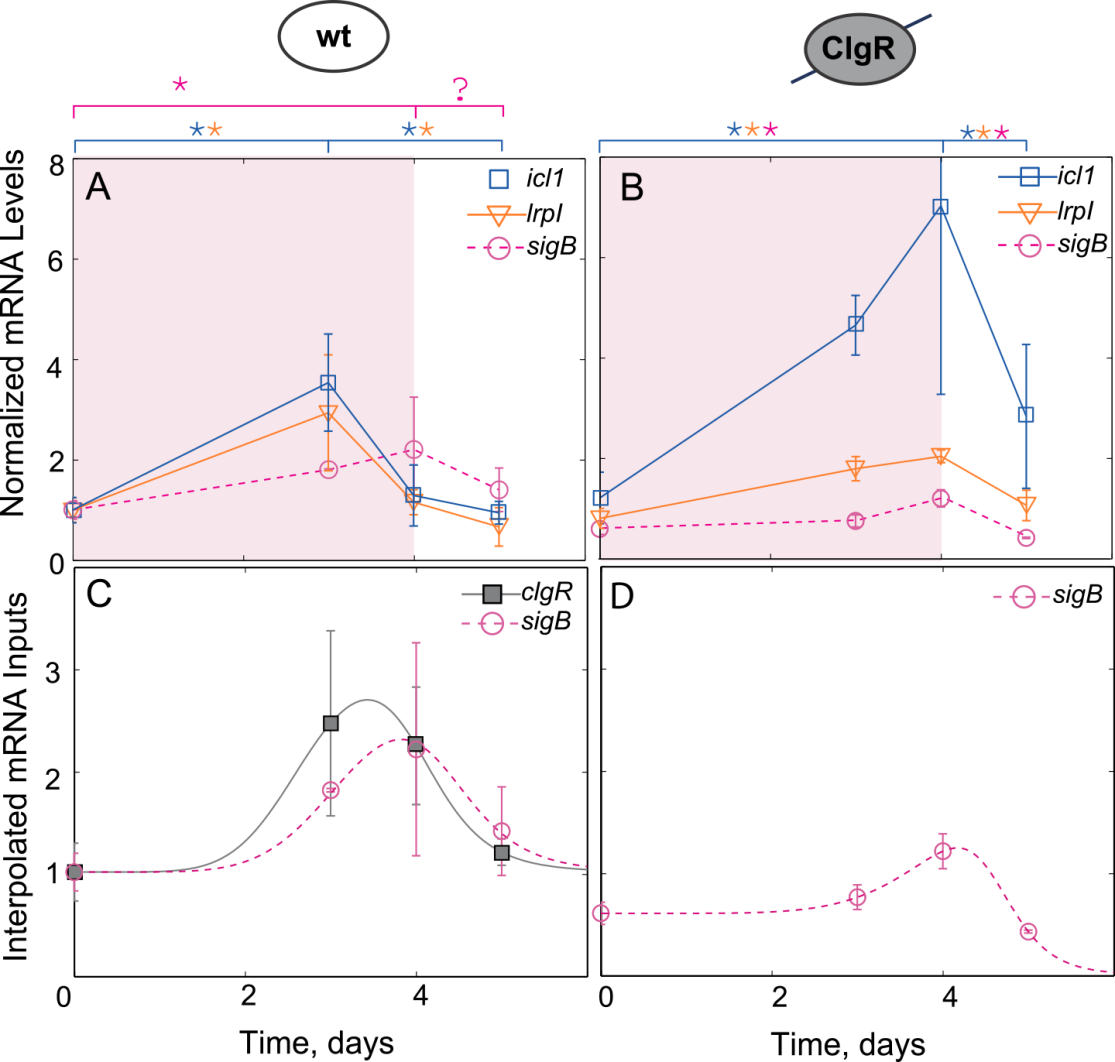
Gene expression measurements reveal a negative regulation of *icl1* through *clgR*. (A,B) Expression measurements for *icl1*, *lrpI* and the accessory sigma factor *sigB* mRNA for wild-type (A) and *clgR* knockout strain (B). (C,D) To simplify the model, the dynamics of *sigB* and *clgR* under hypoxia were interpolated for wild-type (C) and for *clgR* knockout strain (D) with a generalized pulse function and served as inputs into the model (See Methods for details). (*p≤0.05).

Applying the formulated results to the dynamics of *icl1* transcription one can observe that the non-monotonic induction of *icl1* (Fig 1B) is inconsistent with the network diagram proposed in Ref. [12] (Fig 1A) as the hypoxia signal only affects *icl1* via *sigE* (all the directed paths go through *sigE*), and there is no negative parity (inhibiting) path from *sigE* to *icl1*. Nevertheless, the monotonic increase in *sigE* results in non-monotonic dynamics of *icl1*. To further pinpoint the inconsistency, we have repeated the experiments of [12] and additionally measured the mRNA dynamics of all the intermediate species. The results of these measurements are shown in Fig 3A. By applying the monotonic systems theory to these new experimental results we can pinpoint at least two missing negative loops in the previously proposed network architecture (Fig 1A). First, the monotonic increase in *sigE* mRNA leads to non-monotonic increase in its direct target, *sigB*, which increases between days 0 and 4 and then decreases on day 5. We note that the difference between *sigB* mRNA in days 4 and 5 is small and statistical significance of the decrease is questionable (p~0.1). However if the decrease is real, there must be a negative loop in the network that posttranscriptionally regulates activity of σ^E^ or activates transcriptional repressor of *sigB*. However, regardless of this negative loop, another interaction is required to explain the observed *icl1* dynamics. We note that *icl1* starts to decrease after peaking at day 3 (day 3 value is larger than that of day 0 and day 5, p≤0.05); therefore, there must be at least one more negative loop acting downstream of *sigB* transcription. In fact, during the first 4 days of hypoxia, the dynamics of *sigB* is monotonic whereas the dynamics of *lrpI*, its transcriptional target, is not. Furthermore, all directed paths from the hypoxia signal to *lrpI* pass through *sigB*. Therefore, the formulated theorem predicts either a negative feedforward loop between *sigB* and *lrpI* or alternative signaling paths from hypoxia to *lrpI* transcription with a negative parity. In the subsequent sections we focused on uncovering this loop; our approach and workflow is illustrated in S1 Fig.

### ClgR affects network dynamics

The analysis above predicted existence of a negative loop that controls transcription of *lrpI* gene. However, no such loop could be found among known network interactions. One possible way to identify such a loop would be to find a transcriptional repressor of *lrpI* in the σ^B^ or σ^E^ regulons. We therefore examined these regulons for candidate genes with a DNA binding domain and transcriptional regulator function. One such gene is *clgR* (Rv2745c), which encodes a transcription factor regulated by the MprAB-σ^E^ signaling system in response to various stressors, including redox stress, heat shock, and hypoxia [18–20]. ClgR has been reported to induce multiple chaperones and proteases [11,21], including the Clp protease system, which can degrade misfolded proteins and is critical for mycobacterial survival under stress conditions [19]. Since our recent work [9] uncovered a complex network modulating its activity under cell envelope stress, we decided to examine its effect on gene expression in hypoxia.

**Fig 4:**
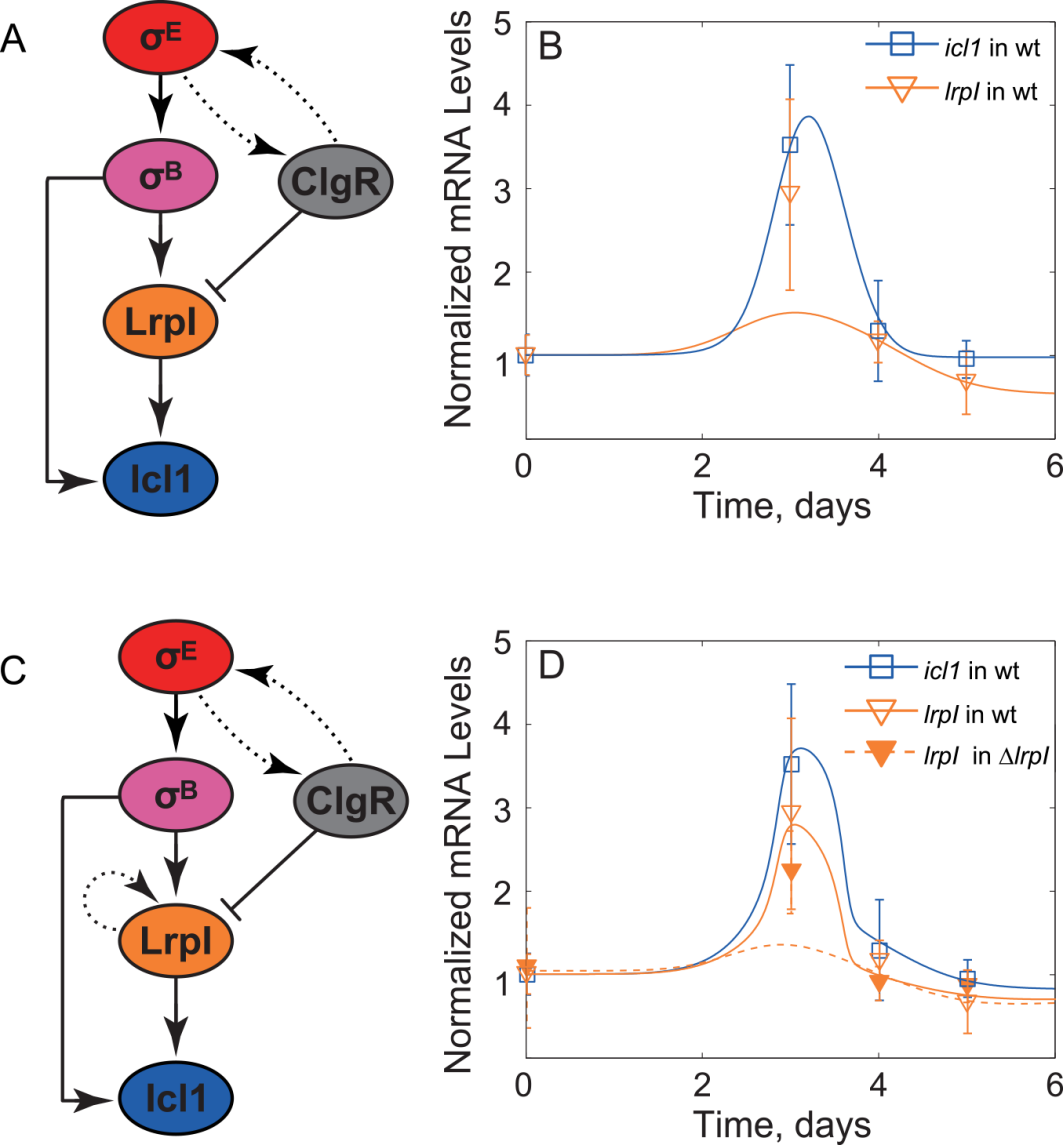
Investigation of repression by ClgR hypothesis. (A) Hypothetical network topology in which ClgR directly downregulated *lrpI* transcription. (B) The predicted dynamics of *icl1* (solid blue line) is in good agreement with experimental observations (blue squares). On the other hand, *lrpI* expression for model (solid orange line) represented in Panel (A) was significantly lower than observed (orange triangles). (C) Hypothetical network that includes additional autoregulation of LrpI in order to explain a non-linear gain. (D) The predicted dynamics (orange and blue solid lines) by the model corresponding to the network topology in panel C shows good agreement with experimental measurements (unfilled triangles and blue squares, respectively). However, the model predicted significantly more attenuation in the *lrpI* mutant (dashed curve) than that of observed in *lrpI* premature codon strain (filled orange triangles).

To this end, we used RT-qPCR to measure the dynamics of *clgR* expression in wild-type cells and examine the expression of *sigB*, *icl1* and *lrpI* in a *clgR* deletion mutant (Δ*clgR*) strain. Deletion of *clgR* resulted in a *sigB* expression pattern that is similar to wild-type but displays about 50% reduction in peak expression (compare Fig 3A and 3B). This is not surprising given that the ClgR-induced protease Clp is responsible for a degradation of the specific anti-σ^E^ factor, RseA [11]. Therefore, the genetic deletion of *clgR* reduces *sigB* transcription by breaking a positive feedback loop that controls σ^E^ activity. In contrast, expression peaks for *icl1* and *lrpI* are increased and shifted to day 4 (Fig 3). We note that in the Δ*clgR* strain non-monotonic induction of *lrpI* follows non-monotonic induction of *sigB* and, therefore, the negative loop controlling *lrpI* transcription may no longer be active. The simplest way to reconcile these results is to hypothesize repression of *lrpI* by ClgR (Fig 4A). Even though ClgR has only been shown to positively regulate its gene products, numerous examples exist of a transcription factor that can act both as activator and repressor of transcription (e.g., the *Bacillus subtilis* master sporulation regulator, Spo0A, which activates about 40 genes and inhibits 81 genes [22]).

### LrpI autoregulation can create non-linear amplification in *lrpI* dynamics

To test whether ClgR-mediated repression of *lrpI* (Fig 4A) can explain the observed dynamics, we first tested this hypothesis by constructing a mathematical model of the network. Given the complexity of *clgR* regulation [9] we included its transcriptional profile as an input to a model by taking the measured data and using an interpolation function to generate a continuous function that follows the observed dynamics (Fig 3C). A similar strategy was used for *sigB* expression (in both wild-type and Δ*clgR* strains)–another input to the network (Fig 3C). These inputs were used in a deterministic, ordinary differential equations model describing the dynamics of σ^B^, ClgR, Icl1 and LrpI protein concentrations and algebraic equations for their respective mRNAs (we assumed the degradation is fast and concentrations are in quasi-steady state). We then performed multidimensional parameter optimization to determine whether the measured dynamics of mRNA can be matched by those produced by the model. It is important to note that while we used parameter optimization as a feasibility check for various network structures, we understand that the data presented is not sufficient to restrict the parameters.

Our simulation results (Fig 4B) showed that the proposed network topology can match the observed data for *icl1* but not for *lrpI*. The failure of this model is due to a non-linear gain between *lrpI* and *sigB* in the data. Indeed, between days 0 and 3 *sigB* mRNA increases about 2-fold whereas *lrpI* mRNA increases over 3-fold. Given that sigma factors bind the core RNA polymerase as a monomer and function non-cooperatively, this result cannot be due to *sigB* mRNA increase. In fact, in steady state we expect σ^B^ protein concentration to scale linearly with *sigB* mRNA and the transcriptional activity of σ^-^factors is usually expected to scale sub-linearly (hyperbolic, Michaelis-Menten-like expression) with their concentration [23]. Therefore, we would expect that, in steady-state, fold-change of σ^B^ -activated mRNA would be lower then fold-change of *sigB* mRNA. Repression by ClgR would further suppress the fold-increase of *lrpI*. The arguments can be formalized and generalized to transient gains as well (see Methods for a rigorous mathematical proof as an application of the corollary of the formulated theorem in S1 Text). Additionally, we demonstrated that even with large variations in the *sigB* and *clgR* input curves, *lrpI* always has a sub-linear amplification (S2 Fig). To resolve this discrepancy, we need a non-linear amplification in the transfer function between *sigB* and *lrpI*. For example, this amplification can be explained if *lrpI* positively regulated its own transcription (Fig 4C). Indeed, incorporating these interactions into our model leads to a good agreement between model predictions and experiments for both wild-type and Δ*clgR* strain (Fig 4D).

### Experimental results rule out *lrpI* autoregulation

To experimentally test autoregulation of *lrpI*, we used an *lrpI* knock-out mutant, in which the *lrpI* open reading frame is interrupted by a transposon insertion. While the strain does not produce functional LrpI protein, it is still possible to quantify the expression of the truncated *lrpI* mRNA by using primers and probes mapping to *lrpI* sequences located upstream of the transposon insertion. In contrast to model predictions, the measured *lrpI* expression in *ΔlrpI* was not statistically significantly different compared to wild type for all time points (Fig 4D). This result rules out *lrpI* autoregulation. Therefore, another factor must be responsible for the non-linear amplification between *sigB* and *lrpI* mRNA. We then investigated an alternative hypothesis, in which post-translational regulation of σ^B^ may lead to a non-linear relationship between *sigB* expression and σ^B^ activity.

### Post-translational modulation of σ^B^ may explain observed dynamics

**Fig 5:**
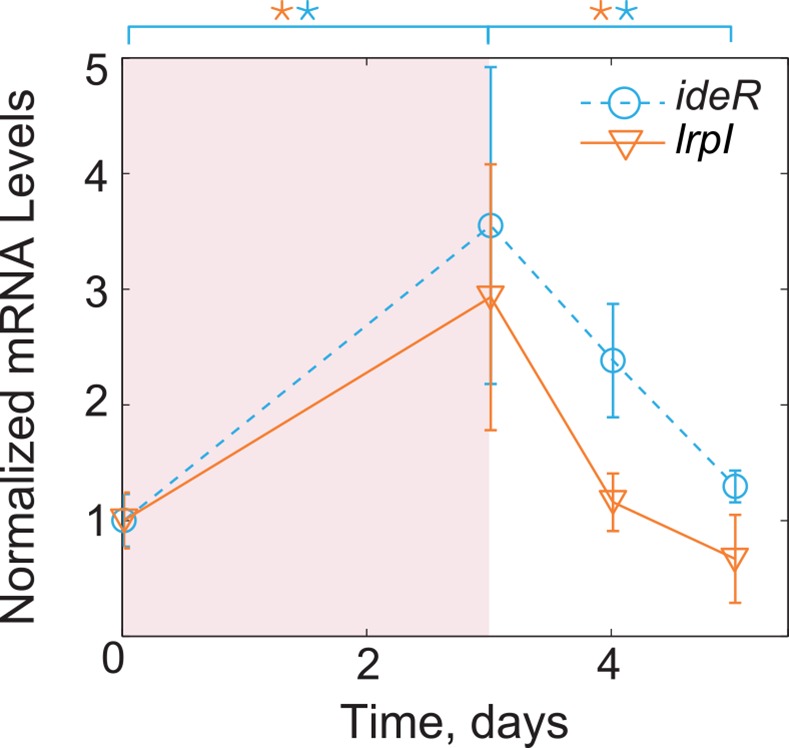
Gene expression measurements for *ideR*. In a well-characterized interaction, σ^B^ acts as the sole transcriptional regulator of *ideR*. mRNA of *ideR* exhibits a non-monotonic response similar to that of *lrpI*, suggesting that the non-monotonic dynamics are a result of a post-translational regulation of σ^B^ activity. (*p≤0.05).

To test the hypothesis that σ^B^ activity is post-translationally regulated, we decided to examine the dynamics of another target. To this end we selected another gene in the σ^B^ regulon, *ideR* (Rv2711), to serve as a reporter for σ^B^ activity. IdeR is a global transcription factor that helps to maintain iron homeostasis and is essential for mycobacterial virulence [24]. Since no other transcriptional inputs have been found (Fig 5A), we assume that *ideR* mRNA represents a surrogate of σ^B^ activity [25]. We therefore used RT-qPCR to quantify how *ideR* expression dynamically changes under hypoxia. Notably, the measured *ideR* dynamics is similar to that of *lrpI* in two important aspects: (i) the fold increase of *ideR* mRNA between days 0 and 3 exceeds that of *sigB* mRNA in the same period (*ideR* has more than 3-fold increase; *sigB* has less than 2-fold increase); (ii) *ideR* peaks at day 3 and decreases at day 4 despite the increase in *sigB* transcription (Fig 5). These results indicate that σ^B^ activity is post-translationally regulated and the missing negative loop must involve post-translational regulation steps.

### Degradation of σ^B^ by Clp may be responsible for σ^B^ activity modulation

Analyzing alternative ways for post-translational downregulation of sigma-factor activity, we identified two theoretical possibilities. One is the downregulation of activity by sequestration of an active form (e.g. by an anti-sigma factor), another is the downregulation of protein level via proteolytic degradation. Since we expect the negative loop to involve ClgR, we hypothesized that ClgR-activated induction of anti-σ^B^ or protease degrading σ^B^ can lead to the observed dynamics.

Anti-sigma factors are ubiquitous across bacterial genera, and have been shown to be present in a diverse array of species [26]. Many of the known sigma factors in *M*. *tuberculosis* have corresponding anti-sigma factors; however, an anti-sigma factor corresponding to the mycobacterial σ^B^ has not yet been identified [27]. Nonetheless, we decided to consider the possibility of a hypothetical anti-σ^B^. While we were unable to definitively exclude the presence of a novel anti-sigma factor B, the inability to fit the models of the various (rather complex) networks to the experimental data (S3 Fig) coupled with the fact that has been no indication of an anti σ^B^ factor in any mycobacterial species [7,27] led us to set aside this hypothesis.

**Fig 6:**
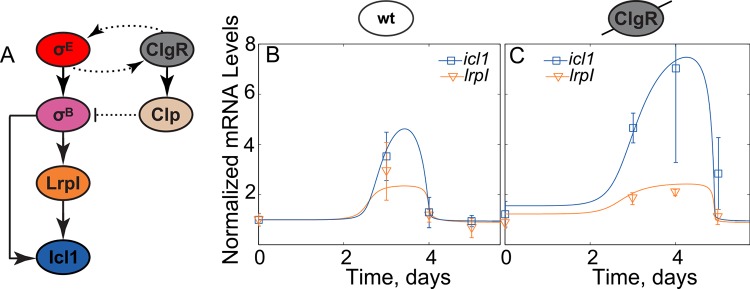
Degradation of σ^B^ by Clp proteases hypothesis. (A) Hypothetical network topology in which ClgR-induced Clp protease degrades σ^B^. The resulting mathematical model shows good agreement between predictions (solid lines) and measurements (squares and triangles) for both wild-type (B) and *clgR*-deletion strain (C). Parameters used in the model correspond to S4 Table.

As previously mentioned, ClgR is known to induce multiple essential protease systems in *M*. *tuberculosis*, which regulate the activity of numerous proteins through selective degradation [11,19,21]. Thus, we hypothesized that one of these protease systems, e.g. Clp, may modulate post-translational σ^B^ activity by selectively degrading the sigma factor (Fig 6A). Incorporation of these interactions into the model leads to simulated dynamics that are in good agreement with the experimental data under a set of physiologically relevant parameters (Fig 6B and 6C; S4 Fig). Indeed, this model is able to replicate the *lrpI*, *ideR* and *icl1* mRNA dynamics both in wild type and *ΔclgR* strains. We further demonstrated that the qualitative output dynamics of the model were very robust to variations in the *sigB* and *clgR* input curves (S5 Fig). These results indicate that the introduction of the previously unknown interaction between Clp and σ^B^ is sufficient to explain all observed dynamics in the *icl1* network.

## Discussion

The combination of traditional molecular genetics and novel high-throughput assays has generated a vast amount of information on the interactions that comprise biochemical networks inside living cells. For example, transcriptional regulatory networks can be obtained from gene-expression analyses such as qPCR, gene expression microarray technology, and RNA-seq, while DNA-protein interactions can be studied by chromatin immunoprecipitation-based (ChIP) measurements. However, regulatory networks are incomplete in even the best studied model systems [28,29]. At the same time, as more dynamical information about the responses of these networks to external perturbations is accumulated, mathematical models can use this data to pinpoint the inconsistencies between observed gene expression dynamics and presumed network topology, and to predict missing interactions [30]. In this context, dynamical systems theories that formulate necessary or sufficient conditions for a given network topology to lead to certain dynamical behaviors are especially useful as these conditions are often independent from detailed reaction mechanisms and kinetic parameters.

Much research exists on the characterization of dynamics of bio-molecular networks by means of their topology. Among many such directions of work, one may mention: (1) the deep theory worked out by Feinberg in the early 1970s based on the idea of deficiency [31], which has been applied to many fields, including T-cell kinetic proofreading models [32] or receptor-ligand pharmaceutical models [33], and is still the subject of a major research effort [34,35]; (2) the use of graph-theoretic ideas based on Petri nets [36]; (3) the theories of cooperative and competitive systems [37]; and (4) methods of commutative algebra and algebraic geometry [38,39].

In this work, we formulated the necessary conditions for an output variable of a dynamical system to non-monotonically vary in time with changing inputs. In simplified terms, a non-monotonic response to monotonic stimulus requires the presence of an indirect negative feedback or an incoherent feedforward loop in the network graph. Even when the exact stimulus dynamics is not known, the comparison of the dynamics of internal components with the topology of the network graph can point to inconsistencies or missing loops.

We applied these results in combination with mathematical modeling and subsequent experimental tests to a network controlling transient upregulation of *icl1*, the gene encoding a glyoxylate shunt enzyme (isocitrate lyase 1), in response to hypoxia in *M*. *tuberculosis*. We found at least one inconsistency in the previously postulated network structure. Our results predict that there should be biochemical interactions that post-transcriptionally downregulate sigma factors σ^B^, and possibly σ^E^. Focusing on the former, we show that this downregulation can be explained by proteolytic degradation of σ^B^ protein by Clp protease system. Our model demonstrates that this interaction is consistent with all measurements for wild-type and genetically modified strains (Fig 6). As ClpP is essential for *M*. *tuberculosis* growth *in vitro* [40], a ClpP knockout mutant could not be created. Thus, this is a major prediction of the model that will be tested in future studies.

The predicted proteolytic degradation of σ^B^ is not unprecedented. In multiple bacterial species, stress-induced alternative sigma factors are proteolytically degraded by *clp* homologs. For example, RpoS (σ^S^) is an enterobacterial sigma factor that is implicated in stationary-phase survival as well as virulence in pathogenic species [8,41–45]. The protease ClpXP has been implicated in the regulation of RpoS in several pathogens, including *S*. *typhimurium* [43,44], and has been directly shown to degrade RpoS in *E*. *coli* [45]. It is known that the mycobacterial σ^B^ and RpoS share an evolutionary relationship [8], which might suggest that proteolytic degradation by ClpP is conserved among some sigma factors. Additionally, ClpP has been shown to degrade σ^B^ of *B*. *subtilis* [46]. Despite sharing a common name, the σ^B^ of *B*. *subtilis* is actually more closely related to the mycobacterial σ^F^ [27]; however, mycobacterial σ^F^ and σ^B^ share a common evolutionary origin [27], again possibly pointing to a potential conserved mechanism for the regulation of stress-induced sigma factors.

What is the significance of the transient activation of *icl1* in hypoxia and the predicted feedforward loops? The hypoxic response can be considered a component of the metabolic adaptation of *M*. *tuberculosis* during infection, both because oxygen may become limiting inside the infected macrophage and because the lung tissue microenvironment becomes hypoxic as a granuloma develops [47–49]. Such adaptation ultimately leads to tubercle bacilli transitioning to a non-replicating/persistent state that is associated with latent infection. It may be argued that the stress-induced activation of adaptive metabolic pathways, such as those involving the activity of the *icl1* product, is transient because the response to stress is followed by slow-down of the mycobacterial metabolic activity. Consequently, no sustained expression of metabolic enzymes such as Icl1 is required. Presumably, the transient surge in *icl1* transcription at day 4 of hypoxia produces sufficient amounts of Icl1 protein until persistence is fully attained. Indeed, the observation that *icl1* is induced transiently also during mouse lung infection supports a physiological role of this dynamics in pathogenesis [12]. If these hypotheses are correct, drugs blocking negative interactions responsible for non-monotonic dynamics could in principle destabilize transitions to latency or trigger reactivation.

The theorem described in this work can be applied to a wide variety of biological systems to help understand the relationship between network topology and dynamics. For example, biochemical adaptation is a topic of wide interest, largely because of its ubiquity in biological systems—adaptation occurs when a step-up input into a biochemical network causes one or more downstream components to transiently increase but then return to the previously maintained steady state. Chemotaxis—the process where a cell moves in response to a chemical signal—is a well-known behavior that exhibits adaptive responses. This behavior is seen in a wide variety of organisms, including bacteria, neutrophils [50] and amoeba [50–52]. The mechanisms whereby these organisms achieve adaptation vary widely, but a well-studied case is chemotaxis in *E*. *coli*—given a step input of a chemoattractant, the rate of ‘tumbling’ will initially sharply decrease, but then return to nearly its original level [53]. Therefore, biochemical adaptation is a specific case where a monotonic input gives rise to a non-monotonic output. We may apply the described theorem to conclude that there must be either an incoherent feedforward loop or a negative feedback loop. Indeed, this observation is recapitulated by previous work by Ma *et al*. that used simulations and parameter sampling to show that there are only two types of networks that can produce robust biochemical adaptation–incoherent feedforward loops and negative feedback loops [54]. Thus, the theorem may help clarify structure-function relationships in well-characterized biochemical networks, as well as predict previously unforeseen inconsistencies in others.

## Methods

### Interpolation of inputs into model

The transcriptional regulation of both *sigB* and *clgR* is complicated and not very well understood [9,23]. To simplify the model, we treated *sigB* and *clgR* as inputs into the system by interpolating the time course of experimentally measured mRNA concentration of both species with a phenomenological function that followed the data trends. The interpolation gives a smooth, continuous function that can approximate species dynamics and can be fed directly into the model. As the wild-type *sigB* and *clgR* data appeared to demonstrate approximately adaptive dynamics, i.e. the time-point at day 5 is approximately the same as day 0, the following pulse function was fitted to the data:

$$m_{B}(t)=1+\left(a_{1}\frac{t^{n}}{a_{2}^{n}+t^{n}}\right)\left(\frac{1}{1+{\left(\frac{t}{a_{3}}\right)}^{m}}\right)$$ (3)

$$m_{ClgR}(t)=1+\left(b_{1}\frac{t^{n}}{b_{2}^{n}+t^{n}}\right)\left(\frac{1}{1+{\left(\frac{t}{b_{3}}\right)}^{m}}\right)$$ (4)

Here *m*, *n* and all *a*_*n*_ / b_n_ are unknown parameters, and *t* is the independent variable (time). The parameters *n* and *m* were fixed to 6 and 12 respectively, while the values *a*_*i*_ and *b*_*i*_ (i = 1,2, 3) were optimized to ensure best fit to the measured data. To this end, experimental data normalized to the value at day 0, and non-linear least-square regression was performed using the MATLAB function *fminsearch* (optimization toolbox) to find the values of the unknown, free parameters. The data point at day 0 was replicated at day 1 for the sole purpose of interpolation as it was observed that relevant gene expression did not change significantly in our experimental set up from days 0–2.

Since the mean value of *sigB* mRNA in the Δ*clgR* strain decreased below its initial level on day 5, a different form of pulse function was fitted to the data:

$$m_{B}(t)=\left(c_{4}+c_{1}\frac{t^{n}}{c_{2}^{n}+t^{n}}\right)\left(\frac{1}{1+{\left(\frac{t}{c_{3}}\right)}^{m}}\right)$$ (5)

In the *clgR* mutant case, the parameters *n* and *m* were fixed to 6 and 18 respectively whereas the values of *c*_*i*_ (i = 1–4) were obtained by non-linear least-square regression as above. Fitted parameter values may be found in S1 Table. It is important to note that the form of the interpolating equations was chosen solely because of their adaptive behavior and ability to change quickly; the form of the equations does not have any biological significance. Therefore, we have also tested the robustness of the model to the precise form of the input by generating a family of input curves.

### Generation of family of *sigB* and *clgR* input curves

In order to better understand both how uncertainty in the *sigB* and *clgR* expression measurements affected the dynamics of downstream nodes, we generated a family of input curves. Random data points were sampled from a normal distribution for each time point of both *sigB* and *clgR*, where the mean and standard deviation corresponded to the measured values. The point at day 0 was replicated to day 1.5, and the point at day 5 was assumed to be near the final steady-state value and thus the point was copied to day 6. These data points were interpolated with a shape-preserving cubic interpolation in MATLAB (*interp1*, method: ‘pchip’). The interpolation curves were then each re-normalized to their initial value. We used the curves as in Figs S2 and S5.

### Dynamical equations

The dynamics of protein concentrations in our model are given by ordinary differential equations that describe the kinetics of protein synthesis (translation) and first-order protein degradation. Here *B* (σ^B^; variable name *B* was used for ease of notation), *LrpI*, *Icl1*, *IdeR*, and *ClgR* represent protein concentrations, and *m*_*x*_ represents the corresponding mRNA concentrations. A description of all parameter symbols may be found in S2 Table. For *B*, concentration of σ^B^ protein, the dynamical equation is of the form:

$$\frac{dB}{dt}=b_{B}m_{B}-k_{degB}B$$ (6)

where *m*_*B*_ is the concentration of *sigB* mRNA. In the same fashion, concentration of ClgR may be described by:

$$\frac{dClgR}{dt}=b_{ClgR}m_{ClgR}-k_{degClgR}ClgR$$ (7)

The dynamics of LrpI and IdeR protein is similarly described:

$$\frac{dLrpI}{dt}=b_{LrpI}m_{LrpI}-k_{degLrpI}LrpI$$ (8)

As we do not have data describing Icl1 and IdeR protein dynamics, and Icl1/IdeR do not affect any other nodes in the network, we did not keep track of Icl1 and IdeR protein in our simulations. To solve these equations we also need equations for mRNA concentrations. As mRNAs usually have a significantly shorter half-life than proteins, we can employ the quasi-steady state approximation and describe mRNA concentration by algebraic equations as function of transcription regulators controlling their expression. For example, *icl1* mRNA concentration (*m*_*icl1*_) is a function of σ^B^ and LrpI concentration:

$$m_{Icl1}=\beta_{icl1}\frac{K_{ILB}^{n_{I}+1}+f_{ILB}(B){\left(LrpI\right)}^{n_{I}}}{K_{ILB}^{n_{I}+1}+(B){\left(LrpI\right)}^{n_{I}}}$$ (9)

Similarly, the concentration of both *ideR* and *lrpI* mRNA is solely a function of σ^B^ concentration.

$$m_{LrpI}=\beta_{LrpI}\frac{K_{LB}+f_{LB}B}{K_{LB}+B}$$ (10)

$$m_{IdeR}=\beta_{IdeR}\frac{K_{RB}+f_{RB}B}{K_{RB}+B}$$ (11)

For the main model, used in Fig 6, a ClgR-induced protease (*Clp*) was introduced, described by the following:

$$m_{Clp}=\beta_{Clp}\frac{K_{CC}+f_{CC}{\left(ClgR\right)}^{n_{CC}}}{K_{CC}+{\left(ClgR\right)}^{n_{CC}}}$$ (12)

$$\frac{dClp}{dt}=b_{Clp}m_{Clp}-k_{degClp}Clp$$ (13)

It was hypothesized that Clp degrades σ^B^, so a new term was added to the equation for dynamics of σ^B^ solely for this model:

$$\frac{dB}{dt}=b_{B}m_{B}-k_{degB}B-k_{cat}\frac{\left(Clp)(B\right)}{K_{M}+B}$$ (14)

The Michaelis-Menten formulation of enzyme kinetics was used here to represent the degradation of σ^B^ by Clp (which assumes that the enzyme-substrate binding is fast). However, similar results were obtained when using the full ODE representation of enzyme kinetics, where *C* is the enzyme-substrate complex, formed between σ^B^ and *Clp*. The formulation is shown below:

$$\frac{dB}{dt}=b_{B}m_{B}-k_{degB}B-k_{f}(B)(Clp)+k_{r}C$$ (15)

$$\frac{dClp}{dt}=b_{Clp}m_{Clp}-k_{degClp}Clp-k_{f}(B)(Clp)+k_{r}C+k_{cat}C$$ (16)

$$\frac{dC}{dt}=k_{f}(B)(Clp)-k_{r}C-k_{cat}C-k_{degC}C$$ (17)

The direct repression of *lrpI* by ClgR with LrpI autoregulation (Fig 4C) is modeled as:

$$m_{LrpI}=\beta_{LrpI}\left(\frac{K_{LB}+f_{LB}B}{K_{LB}+B}\right)\left(\frac{K_{LL}^{n_{L}}+f_{LL}{LrpI}^{n_{L}}}{K_{LL}^{n_{L}}+{LrpI}^{n_{L}}}\right)\left(\frac{1}{1+{\left(\frac{ClgR}{K_{LC}}\right)}^{n_{c}}}\right)$$ (18)

For the model without *lrpI* autoregulation (Fig 4A and 4B), we use f_LL_ = 1 and as a result:

$$m_{LrpI}=\beta_{LrpI}\left(\frac{K_{LB}+f_{LB}B}{K_{LB}+B}\right)\left(\frac{1}{1+{\left(\frac{ClgR}{K_{LC}}\right)}^{n_{c}}}\right)$$ (19)

For the simulations shown on S3A Fig, we use Eqs (7–9 and 19) for ClgR, LrpI, IdeR, Icl1 and use an additional equation for a hypothetical anti-sigma factor (*A*), adds new terms of binding and dissociation to σ^B^ dynamics to describe the sequestration of *B* with *A* to form a complex (*C*_*2*_):

$$\frac{dB}{dt}=b_{B}m_{B}-k_{degB}B-k_{f}BA+k_{r}C_{2}$$ (20)

*A* is the (free) concentration hypothetical anti-sigma factor and *A*_*T*_ is the total amount of anti-sigma factor (bound + unbound). As we assume that the total amount of A (*A*_*T*_) is constant, it was treated a system parameter. The complex then follows the following dynamics:

$$\frac{dC_{2}}{dt}=k_{f}B∙A-k_{r}C_{2}-k_{degC}C_{2}$$ (21)

At quasi-steady state, we use the following expression:

$$A=\frac{(k_{r}+k_{degC})A_{T}}{k_{r}+k_{degC}+k_{f}B}$$ (22)

The case where *A* is regulated by ClgR was also explored (refer to S3C Fig). Most equations remain the same, except the total amount of *A* is no longer treated a system parameter, and the pseudo-steady state approximation is not applied to *C*. The following describe the mRNA and protein of A:

$$m_{A}=\beta_{A}\frac{K_{AC}^{n_{A}}+f_{AC}{\left(ClgR\right)}^{n_{A}}}{K_{AC}^{n_{A}}+{\left(ClgR\right)}^{n_{A}}}$$ (23)

$$\frac{dA}{dt}=b_{A}m_{A}-k_{degA}A$$ (24)

Similarly, the case where σ^B^ activated transcription of the hypothetical sigma factor was also investigated (refer to S3B Fig); simply replace *ClgR* with *B* and set *n*_*A*_ to 1 in Eq 24.

### Relationship between the relative changes of *lrpI* and *sigB* mRNA

Below we demonstrate that regardless of the parameter values and for any monotonically increasing *m*_*ClgR*_(*t*) > 0 and *m*_*B*_(*t*) > 0, the solution dynamical system consisting of differential Eqs (6–8) and algebraic Eq (19) starting from steady-state at t = 0 would be subject to the following condition:

$$\frac{m_{B}(t)}{m_{B}(0)}\geq \frac{m_{LrpI}(t)}{m_{LrpI}(0)}$$ (25)

To prove this, we first note that with monotonically increasing *m*_*ClgR*_(*t*) in Eq (7) we can conclude that

ClgR(t)≥ClgR(0) (26)

Now consider alternative dynamical system for which ${\tilde{m}}_{ClgR}(t)={\tilde{m}}_{ClgR}(0)$ and consequently $C\tilde{lg}R(t)=C\tilde{lg}R(0)$. Here and below ~ denotes variables of an alternative system. Now from Eq (19) we can see that

$$m_{LrpI}(t)\leq {\tilde{m}}_{LrpI}(t)\;\text{and}\;m_{LrpI}\left(0\right)={\tilde{m}}_{LrpI}\left(0\right)$$ (27)

As the last term in Eq (19) is a decreasing function of ClgR and the rest of the terms are the same in original and alternative system. We also note that the alternative system no longer has negative loop between the input, *m*_*B*_(*t*), and the output, ${\tilde{m}}_{LrpI}(t)$. Therefore, we can apply the result on the steady state gain (S1 Text) to conclude that

$${\tilde{m}}_{LrpI}(t)\leq \beta_{LrpI}\left[\frac{K_{LB}+f_{LB}b_{B}m_{B}(t)/k_{degB}}{K_{LB}+b_{B}m_{B}(t)/k_{degB}}\right]\left(\frac{1}{1+{\left(\frac{ClgR(0)}{K_{LC}}\right)}^{n_{c}}}\right)$$ (28)

Here the right-hand side is i/o steady state response of the system deduced from Eqs (6) and (18) (*G(u(t)* in the notation of the S1 Text (page 5), with *m*_*B*_(*t*) = *u*(*t*) as an input). We note that the expression in the square brackets is a sublinear function of *m*_*B*_(*t*) and therefore

$$\frac{K_{LB}+f_{LB}b_{B}m_{B}(t)/k_{degB}}{K_{LB}+b_{B}m_{B}(t)/k_{degB}}\leq \frac{m_{B}(t)}{m_{B}(0)}\frac{K_{LB}+f_{LB}b_{B}m_{B}(0)/k_{degB}}{K_{LB}+b_{B}m_{B}(0)/k_{degB}}$$ (29)

Now by combining these results we conclude

$$\frac{m_{LrpI}(t)}{m_{LrpI}(0)}\leq \frac{{\tilde{m}}_{LrpI}(t)}{{\tilde{m}}_{LrpI}(0)}\leq \frac{m_{B}(t)}{m_{B}(0)}$$ (30)

We note that for t = 3 days we have $\frac{m_{B}(t)}{m_{B}(0)}∼2$and $\frac{m_{LrpI}(t)}{m_{LrpI}(0)}∼3$ contradicting this inequality. Thus the model without autoregulation of can never match the observed fold-change in *m*_*LrpI*_

### Simulations

The formulated system of equations for each model was analyzed using a number of tools and functions in MATLAB 2013(a). Solution of the system of ODEs was obtained with ODE15s solver, as the parameter variation during optimization may cause system stiffness. Each case was run using 500 different initial parameter sets, by setting the initial parameters to a set of random numbers generated through the *RandStream* function in MATLAB, seeded with 'mt19937ar’. All parameter optimization simulations were run on the Rice Shared Tightly-Integrated Cluster (STIC).

### Parameter optimization

Parameter fitting was employed in order to test the compatibility of the proposed network topologies with the experimental data. If there is a set of parameters that allows the dynamical equations describing the system to sufficiently replicate the experimental data, then the network topology may be feasible. In order to fit system parameters to the data, the parameters were varied in order to attempt to minimize the deviation of the numerical solution of the dynamical system from the experimental data (least squares). All fits used unweighted least squares, except the wild type *ideR* and *icl1* data, which was weighted by the standard deviations of each data point because the data point at day 3 and 4 respectively had particularly large standard deviations. Parameter optimization was performed using particle swarm optimization (*pso*), a metaheuristic, constrained optimization routine. The same implementation of *pso* for MATLAB was used for all aforementioned cases [55]. The *pso* algorithm was set to have a maximum generation number of 5000 and a population size equal to the number of free parameters. The parameter constraints are delineated in S3 Table, and the parameters corresponding to the fit on Fig 6 are shown in S4 Table.

### Statistical tests

In order to avoid assumptions regarding the underlying distribution of the data, a Wilcoxon rank-sum test (in MATLAB) was used to evaluate statistical significance for all tests. A one-tailed test was employed to evaluate if the peak expression was larger than both the first and last data points for the dynamics of each gene product (*sigB*, *clgR*, *lrpI*, *icl1*, *ideR*). A two-tailed test was used to evaluate if there was a significant difference between *lrpI* expression in wt and Δ*lrpI* strains for all time points.

### Bacterial strains, reagents and media, and growth conditions

*M*. *tuberculosis* mutants with deletion of *sigE*, *sigB*, or *clgR*, were previously reported [56–58] and a transposon-insertion mutants in gene *rv0465c* was obtained from the BEI repository [12,59]. The gene numbering of the *M*. *tuberculosis* genome is presented according to the system of Cole et al. [60]. Aerated and hypoxic cultures of *M*. *tuberculosis* were grown in Dubos Tween-albumin broth (Becton Dickinson) or Middlebrook (MB) 7H10 (solid medium) (Difco) supplemented with 0.05% Tween 80, 0.2% glycerol, and 10% ADN (2% glucose, 5% bovine serum albumin [BSA; Sigma], 0.15 M NaCl). Aerated liquid cultures of *M*. *tuberculosis* were grown in 25-ml tubes at 37°C with magnetic-bar stirring at 450 rpm. Hypoxic cultures of *M*. *tuberculosis* were grown as described below [12]. Bacilli growth was monitored by measuring optical density or enumeration of colony forming units. Aliquots of cultures were harvested at selected time points and processed for RNA extraction. We note that we observed no growth defects in the mutants, and the optical densities across the time-courses were nearly identical for all strains used (S6 Fig).

### Gradual oxygen depletion model

When *M*. *tuberculosis* cultures reached OD580 of 0.4 (mid-log phase), they were diluted to an OD580 of 0.004. Gradual oxygen depletion was achieved by incubating 17 ml-aliquots of diluted culture in 25-ml culture tubes containing a magnetic stirring bar. This design results in a ratio of headspace air to medium of 0.5, in accordance with the classical method established by Wayne and Hayes [61].

### RNA extraction and enumeration of transcripts

RNA extraction and enumeration of bacterial transcripts were performed as described previously [12,62,63]. Briefly, total RNA was purified using TRI reagent (Molecular Research Center, Cincinnati, OH) according to the manufacturer's protocol. Reverse transcription was performed with random hexameric primers and ThermoScript reverse transcriptase (Life technology, Carlsbad, CA). Transcripts were enumerated by real time PCR in a Stratagene Mx4000 thermal cycler (Agilent Technologies), using gene-specific primers, and molecular beacons (refer to S5 Table). Transcript numbers were normalized to 16S rRNA copy number of *M*. *tuberculosis*, as described previously [12,62]. To compare with simulations, all time course qRT-PCR data in the wild-type strain were normalized to the first data point (time 0), whereas all data from mutant strains were normalized to the first time point of the corresponding wild type strain.

## Supporting Information

S1 Fig Block-diagram summary of approach shown in this paper. (PDF)

S2 Fig Repression of *lrpI* by ClgR alone cannot account for nonlinear amplification of *lrpI*. (A) A family of *sigB* and *clgR* input curves (100 pairs) was created (see Methods for details); for each pair of input curves, optimization was repeated for the model shown in Fig 4A and 4B. Amplification of *lrpI* was plotted for all 100 simulations, all of which resulted in sublinear *lrpI* amplification at day 3, in stark contrast to the experimental data. The blue circle represents the mean experimentally determined amplification of *lrpI* and the error bars show ±one standard deviation of both *lrpI* and *sigB* at day 3
(PDF)

S3 Fig Models with a hypothetical anti-sigma factor B is were not able to replicate experimental data. Several models were constructed where *lrpI* is directly downregulated by ClgR and a hypothetical anti-σ^B^ factor, A, was introduced that was either (A) constitutively expressed, (B) regulated by σ^B^, or (C) regulated by ClgR. The predicted dynamics (optimal parameter sets, solid lines) do not replicate the experimental data (triangles and squares) in both the wild type (D-F) and ClgR mutant strain (G-I), as well as the wild type *ideR* dynamics (J-L).
(PDF)

S4 Fig Predicted *ideR* dynamics are consistent with experimental data in Clp model. Additional fitting results to the model shown in Fig 6. As shown, the predicted *ideR* mRNA dynamics agree well with the experimental data.
(PDF)

S5 Fig Qualitative dynamics of *icl1* are robust to variations in the input functions. (A) A family of *sigB* and *clgR* input curves (100 pairs) was created (see Methods for details), and (B) the dynamics of *icl1* were modeled with the same network and parameters as Fig 6 (S4 Table); the bold line represents median *icl1* expression at each time point. However, there was no indication in the data that either *sigB* or *clgR* mRNA decreased below its initial value after day 0, so all curve pairs where either *sigB* or *clgR* fell below 1 were excluded in C; the *icl1* curves corresponding to the non-excluded input curves are shown in D.
(PDF)

S6 Fig Growth curves of all *M*. *tuberculosis* strains examined in this work. (PDF)

S1 Table Input interpolation parameter values. (PDF)

S2 Table Description of parameters. (PDF)

S3 Table Parameter ranges. (PDF)

S4 Table Optimized parameter values corresponding to Fig 6. (PDF)

S5 Table Primers (Fwd and Rev) and molecular beacons (MB). (PDF)

S1 Text Proof of Theorem. (PDF)

S1 Data All experimental data used in this work. (XLSX)

S1 Code Model corresponding to Fig 6 implemented in MATLAB. (M)
