# Supplementary figures and images for: Non-monotonic Response to Monotonic Stimulus: Regulation of Glyoxylate Shunt Gene-Expression Dynamics in Mycobacterium tuberculosis
Source: PLoS Comput Biol. 2016 Feb 22;12(2):e1004741. doi: 10.1371/journal.pcbi.1004741 (PMC4762938; doi:10.1371/journal.pcbi.1004741)

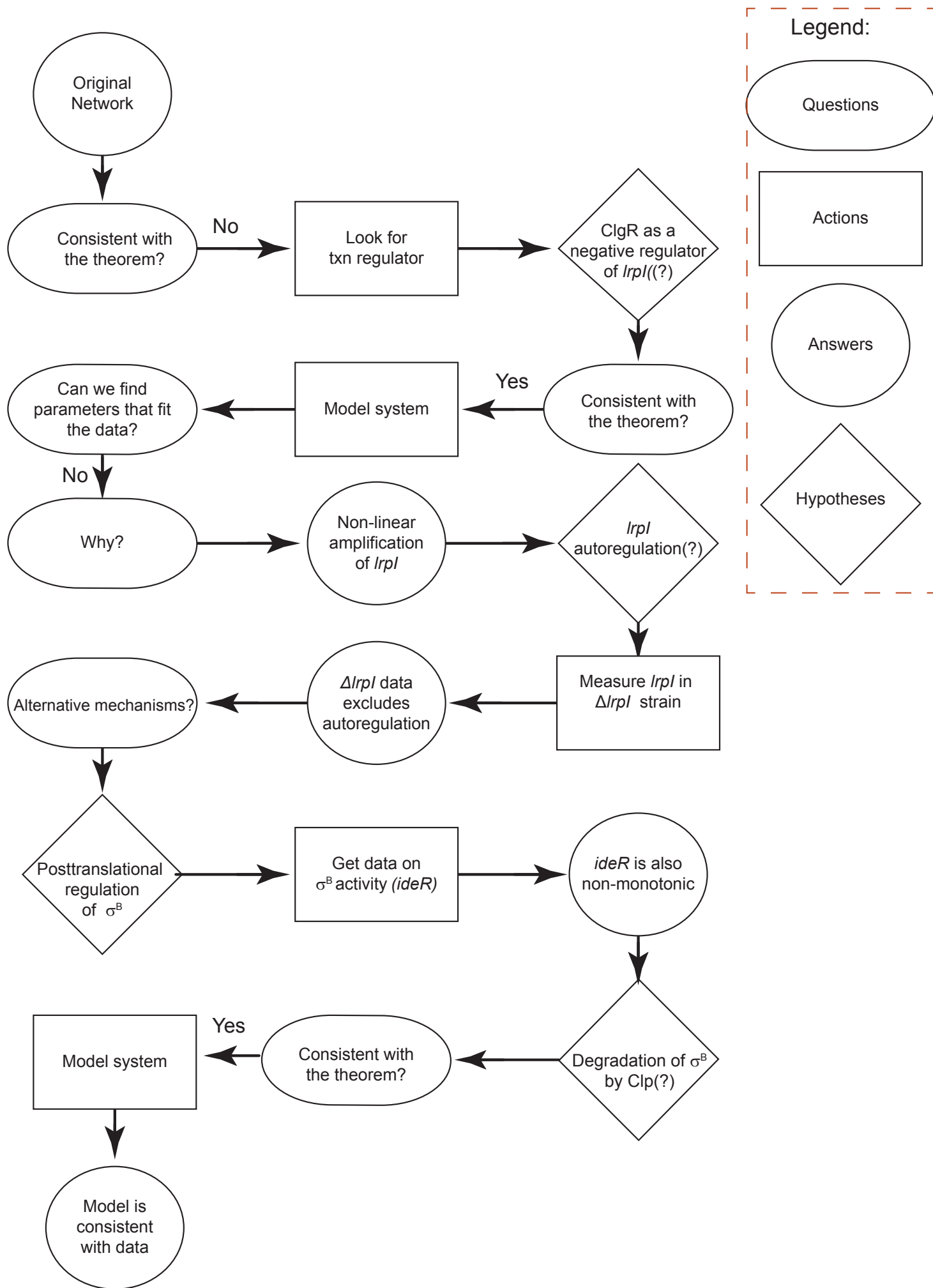

Supplement: S1 Fig — (PDF) [file pcbi.1004741.s001.pdf]

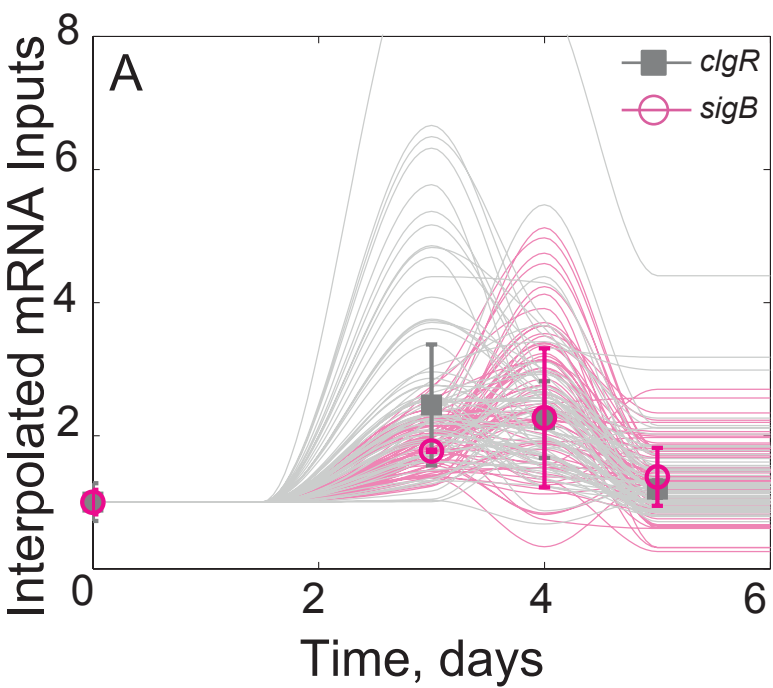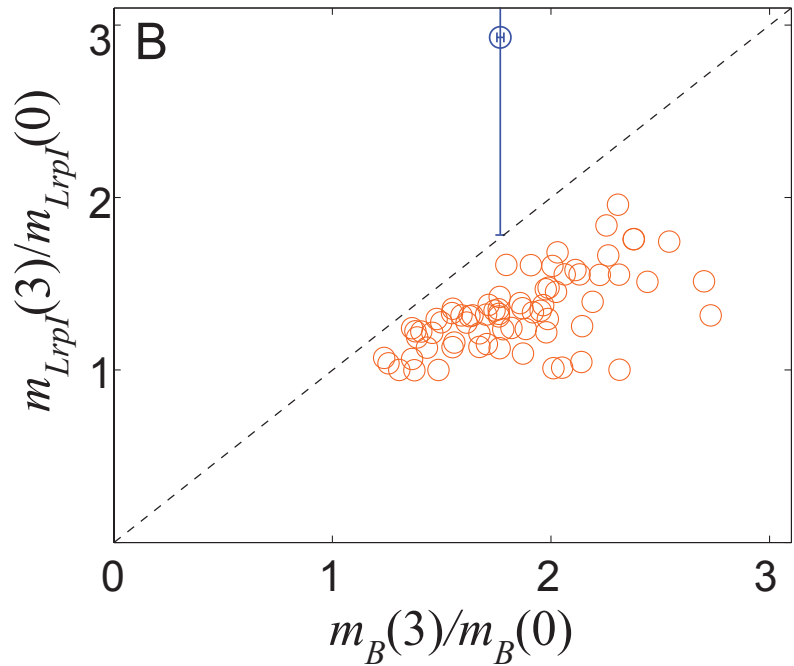

Supplement: S2 Fig — (A) A family of sigB and clgR input curves (100 pairs) was created (see Methods for details); for each pair of input curves, optimization was repeated for the model shown in Fig 4A and 4B. Amplification of lrpI was plotted for all 100 simulations, all of which resulted in sublinear lrpI amplification at day 3, in stark contrast to the experimental data. The blue circle represents the mean experimentally determined amplification of lrpI and the error bars show ±one standard deviation of both lrpI and sigB at day 3 (PDF) [file pcbi.1004741.s002.pdf]

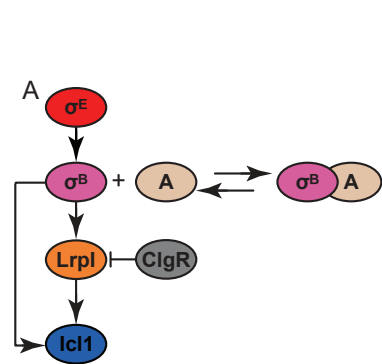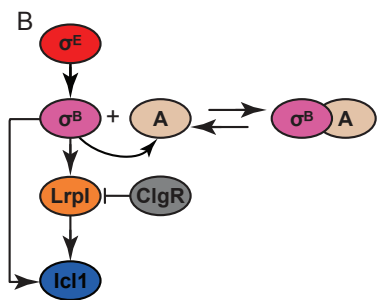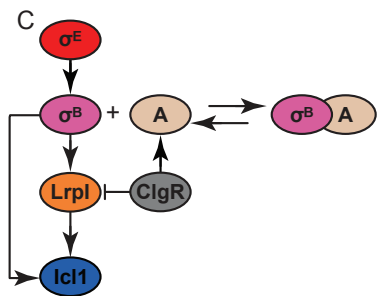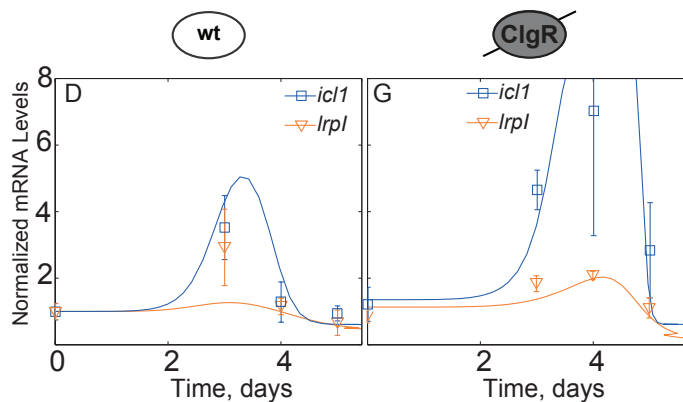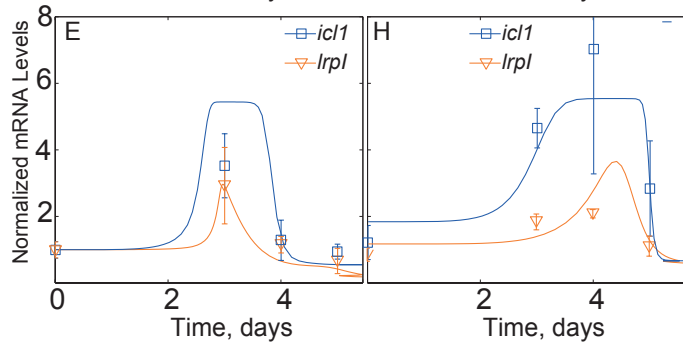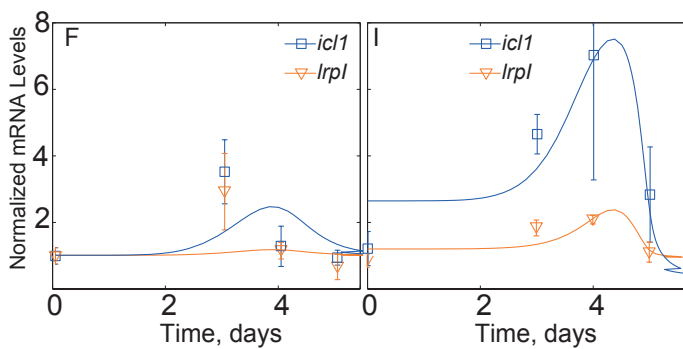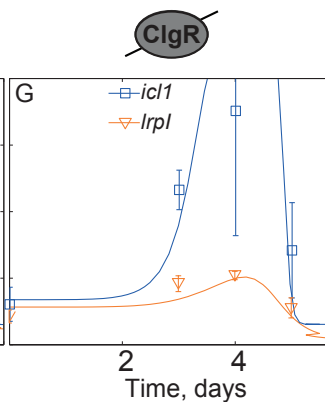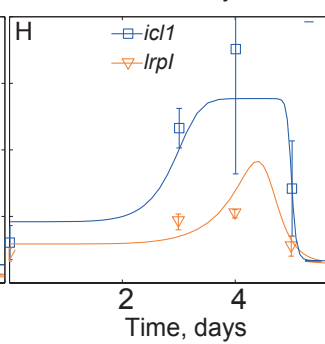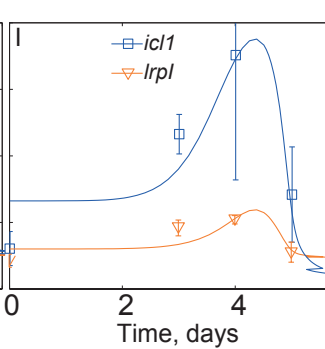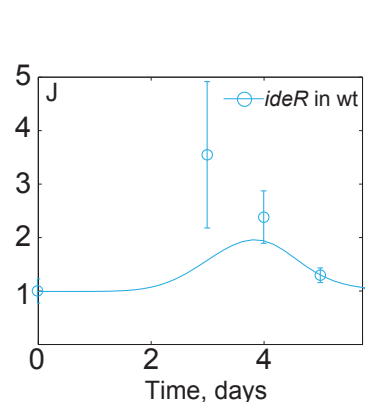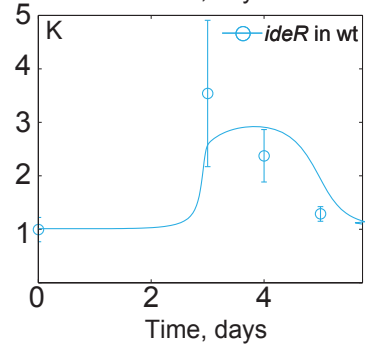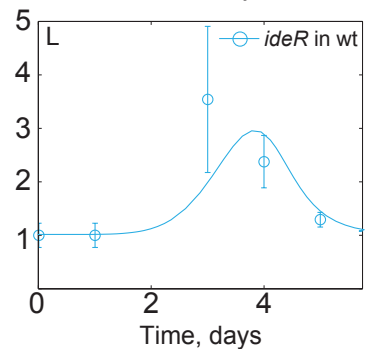

Supplement: S3 Fig — Several models were constructed where lrpI is directly downregulated by ClgR and a hypothetical anti-σB factor, A, was introduced that was either (A) constitutively expressed, (B) regulated by σB, or (C) regulated by ClgR. The predicted dynamics (optimal parameter sets, solid lines) do not replicate the experimental data (triangles and squares) in both the wild type (D-F) and ClgR mutant strain (G-I), as well as the wild type ideR dynamics (J-L). (PDF) [file pcbi.1004741.s003.pdf]

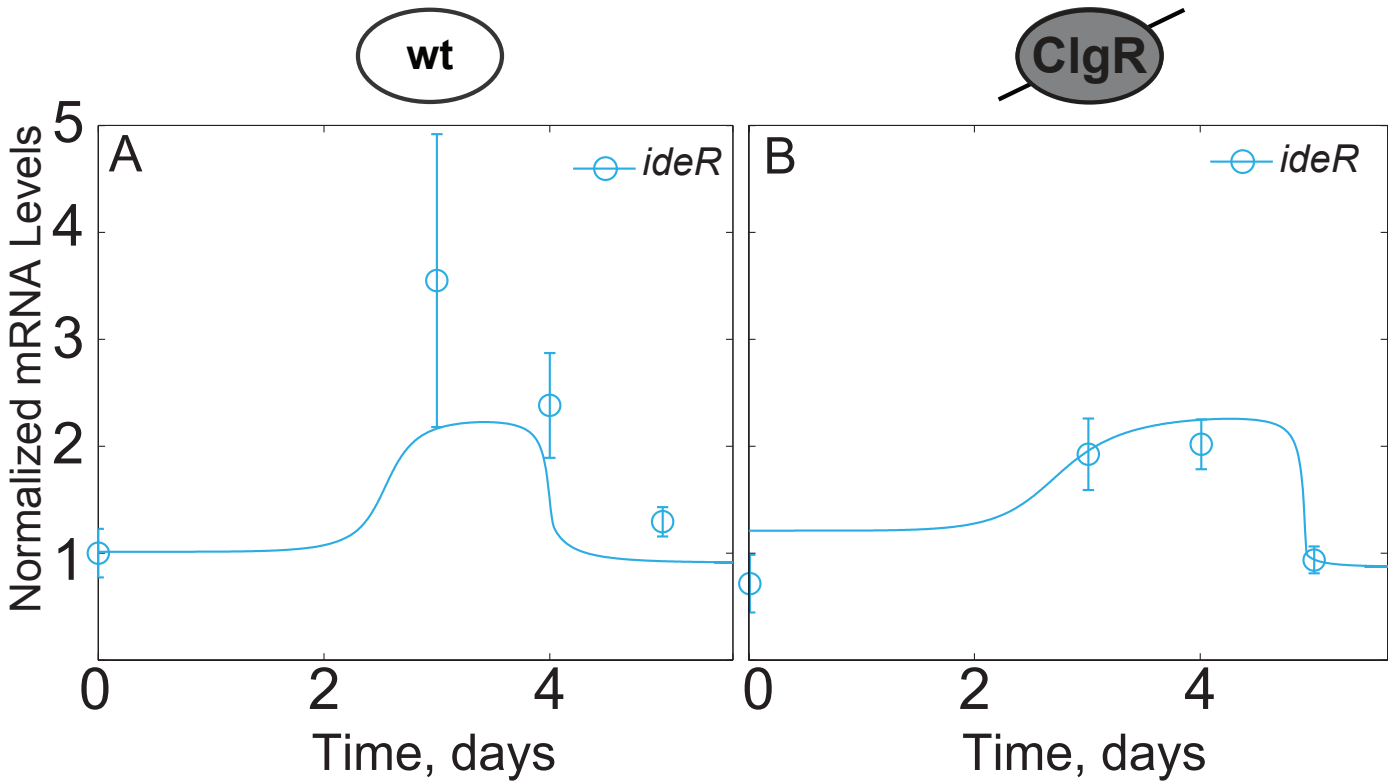

Supplement: S4 Fig — Additional fitting results to the model shown in Fig 6. As shown, the predicted ideR mRNA dynamics agree well with the experimental data. (PDF) [file pcbi.1004741.s004.pdf]

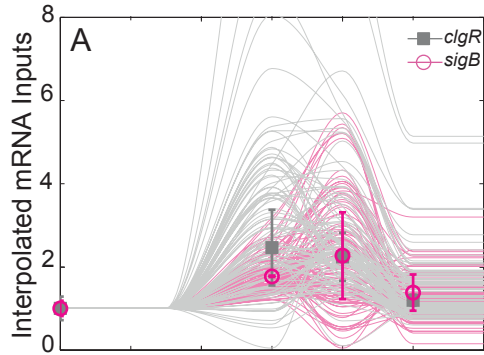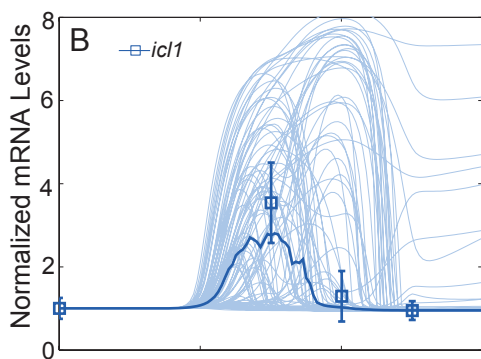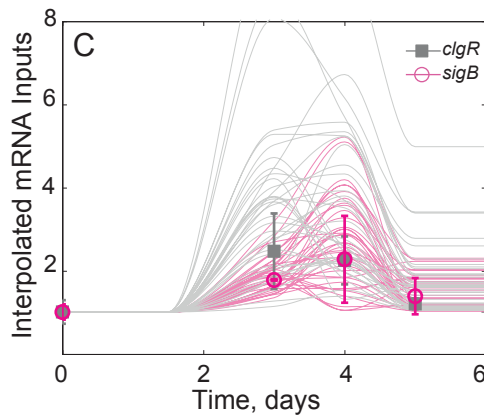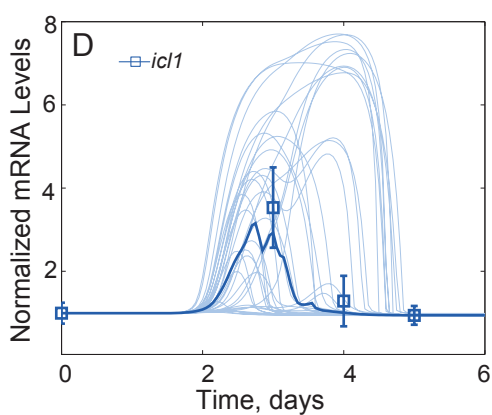

Supplement: S5 Fig — (A) A family of sigB and clgR input curves (100 pairs) was created (see Methods for details), and (B) the dynamics of icl1 were modeled with the same network and parameters as Fig 6 (S4 Table); the bold line represents median icl1 expression at each time point. However, there was no indication in the data that either sigB or clgR mRNA decreased below its initial value after day 0, so all curve pairs where either sigB or clgR fell below 1 were excluded in C; the icl1 curves corresponding to the non-excluded input curves are shown in D. (PDF) [file pcbi.1004741.s005.pdf]

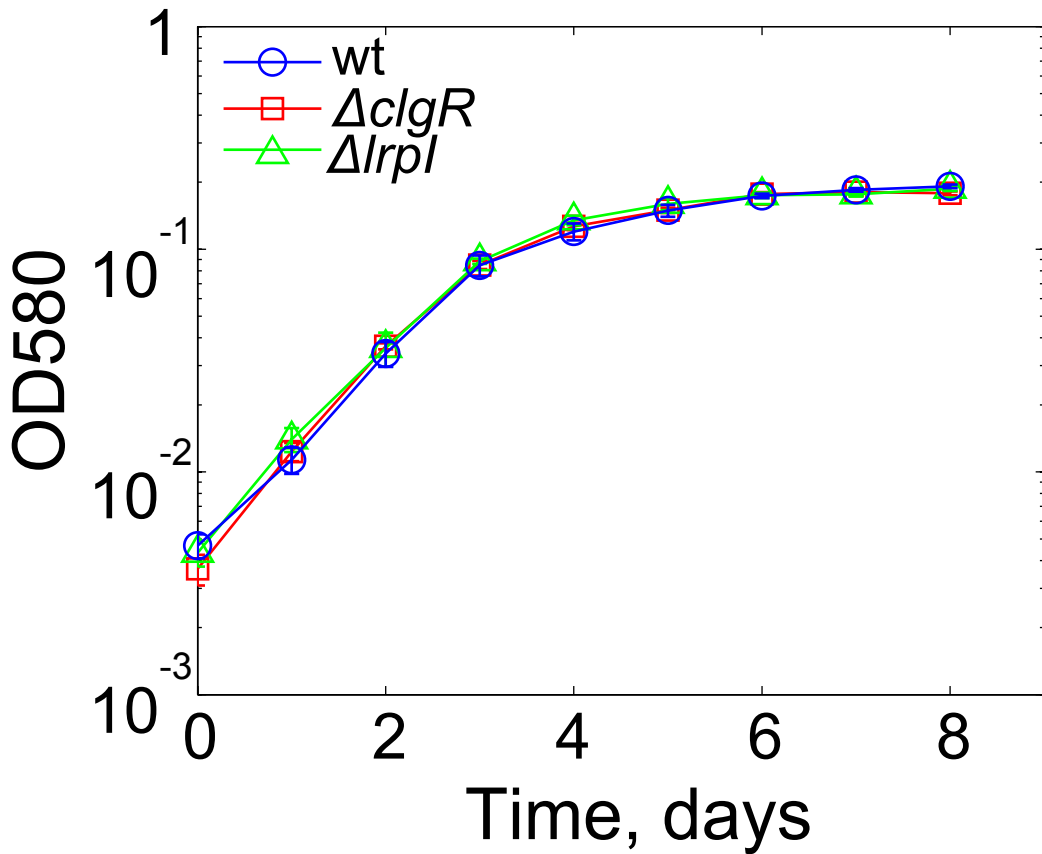

Supplement: S6 Fig — (PDF) [file pcbi.1004741.s006.pdf]
