# Supplementary material for: Non-monotonic Response to Monotonic Stimulus: Regulation of Glyoxylate Shunt Gene-Expression Dynamics in Mycobacterium tuberculosis
Source: PLoS Comput Biol. 2016 Feb 22;12(2):e1004741. doi: 10.1371/journal.pcbi.1004741 (PMC4762938; doi:10.1371/journal.pcbi.1004741)
Supplement: S1 Table — (PDF) [file pcbi.1004741.s007.pdf]

**Table S1: Input interpolation parameter values**			
	a_n	b_n	c_n
1	2.137	2.399	2.598
2	3.296	2.764	4.732
3	4.434	4.095	4.631
4	--	--	0.603
