# Supplementary material for: Non-monotonic Response to Monotonic Stimulus: Regulation of Glyoxylate Shunt Gene-Expression Dynamics in Mycobacterium tuberculosis
Source: PLoS Comput Biol. 2016 Feb 22;12(2):e1004741. doi: 10.1371/journal.pcbi.1004741 (PMC4762938; doi:10.1371/journal.pcbi.1004741)
Supplement: S2 Table — (PDF) [file pcbi.1004741.s008.pdf]

Table S2: Description of Parameters	
**Parameter**	**Description**
β	Ratio of basal transcription rate to mRNA degradation rate
b	Protein translation rate
n	Kinetic order
f	Amplification gain
k_{deg}	Protein degradation rate
K	Equilibrium dissociation constant
k_f	Rate of forward binding reaction
k_r	Rate of reverse binding reaction
k_{cat}	Rate of catalytic step in degradation reaction
K_M	Michaelis-menten constant
