# Supplementary material for: Non-monotonic Response to Monotonic Stimulus: Regulation of Glyoxylate Shunt Gene-Expression Dynamics in Mycobacterium tuberculosis
Source: PLoS Comput Biol. 2016 Feb 22;12(2):e1004741. doi: 10.1371/journal.pcbi.1004741 (PMC4762938; doi:10.1371/journal.pcbi.1004741)
Supplement: S3 Table — (PDF) [file pcbi.1004741.s009.pdf]

Table S3: Parameter Ranges		
**Parameter**	**Lower Limit**	**Upper Limit**
f	1	100
n	0.1	10
K	1	1000
All other parameters	0.001	1000
