# Supplementary material for: Non-monotonic Response to Monotonic Stimulus: Regulation of Glyoxylate Shunt Gene-Expression Dynamics in Mycobacterium tuberculosis
Source: PLoS Comput Biol. 2016 Feb 22;12(2):e1004741. doi: 10.1371/journal.pcbi.1004741 (PMC4762938; doi:10.1371/journal.pcbi.1004741)
Supplement: S4 Table — (PDF) [file pcbi.1004741.s010.pdf]

Table S4: Optimized Parameter Values Corresponding to Fig. 6	
**Parameter Name**	**Parameter Value**
f_{LB}	2.85
f_{RB}	2.67
f_{ILB}	9.38
f_{CC}	53.0
K_{LB}	4.25
K_{ILB}	18.1
K_{RB}	3.69
K_{CC}	547
n_I	5.98
n_{CC}	3.75
β_{LrpI}	0.057
β_{Icl1}	2.60
β_{Clp}	619
β_{IdeR}	0.0051
b_{Clp}	7.46
b_B	986
b_{ClgR}	3.12
b_{LrpI}	0.36
k_{degB}	9.05
$k_{degClgR}$	1.65
$k_{degLrpI}$	0.0015
k_{degClp}	0.11
k_{cat}	0.015
K_M	0.10
