# Supplementary material for: Non-monotonic Response to Monotonic Stimulus: Regulation of Glyoxylate Shunt Gene-Expression Dynamics in Mycobacterium tuberculosis
Source: PLoS Comput Biol. 2016 Feb 22;12(2):e1004741. doi: 10.1371/journal.pcbi.1004741 (PMC4762938; doi:10.1371/journal.pcbi.1004741)
Supplement: S5 Table — (PDF) [file pcbi.1004741.s011.pdf]

Table S5: Primers (Fwd and Rev) and molecular beacons (MB)	
icl1	Fwd: GGCCCTCTTCGGTGGAAC<br>Rev: CCAGAACCAGATGAGCGCGTAT<br><br>MB: ACGCCCGGGGCTACACCGCGACCAA GGGCGT
lrpI	Fwd: GTTCTTTGCCTCCCAGGACGACA<br>Rev: GCCACTTCATGCGGGTCGATG<br><br>MB: CGCACGTTGCCGAACTCAGGGAGGTGACCGTGCG
sigE	Fwd: ACGACTTGCCAACTTATTGCAG<br>Rev: GGATGAGACATGCTGGTCGGAC<br><br>MB: CGCACGATATCACGACCATCACGACC TTGCGTGCG
sigB	Fwd: ACAAGCCGGGTTGACAGCGATCT<br>Rev: TGCGCTTGGCCAGTTCGACTTC<br><br>MB: CGCACGCTCAAAGCCCCGCGGCGGACGTGCG
clgR	Fwd: TGAGCCTCGGGTATCTGTCGG<br>Rev: AAGGCGCTCTTGACGCGCCAT<br><br>MB: ACGGGGCGAGCTGCTCAGTGCGATTTGTACCCCGT
ideR	Fwd: AACGCACGAGTAACCGTCGAAAC<br>Rev: TCAGACTTTCTCGACCTTGACCGC<br><br>MB: CCCCGGCGGCGTGACCATCGTCATCCCGGGG
16S rRNA	Fwd: ATGACGGCCTTCGGGTTGTAA<br>Rev: CGGCTGCTGGCACGTAGTTT<br><br>MB: CCCCGCCGACGAAGGTCCGGGTTCTCGCGGGG
