# Supplementary material for: Non-monotonic Response to Monotonic Stimulus: Regulation of Glyoxylate Shunt Gene-Expression Dynamics in Mycobacterium tuberculosis
Source: PLoS Comput Biol. 2016 Feb 22;12(2):e1004741. doi: 10.1371/journal.pcbi.1004741 (PMC4762938; doi:10.1371/journal.pcbi.1004741)
Supplement: S1 Text — (PDF) [file pcbi.1004741.s012.pdf]

1 Proof of Theorem

We consider systems of the general form

$$\dot{X} = F(X, u),$$

where states

$$X(t) = (X_1(t), \ldots, X_n(t))$$

evolve on an open subset $X \subseteq \mathbb{R}^n$ and

$$F = (F_1, \ldots, F_n)^T$$

is a continuously differentiable function $F : X \times U \to \mathbb{R}^n$, where $U \subseteq \mathbb{R}$ is the set where inputs

$$u : [0, \infty) \to \mathbb{R}$$

take values. Dot indicates derivative with respect to time. As a function of time, the input signal $u(t)$ is assumed to be continuous or at most has a discrete set of discontinuities (for example, a step function). We assume that the partial derivatives

$$\frac{\partial F_j}{\partial X_i}(X, u)$$

and

$$\frac{\partial F_j}{\partial u}(X, u)$$

have the same sign (either ≥ 0 or ≤ 0) for all $(X, u) \in X \times U$. For those derivatives that are not identically zero, we write φ_{ij} for the sign (± 1)

$$\varphi_{ij} := \text{sign}\frac{\partial F_j}{\partial X_i}(X, u)$$

and γ_i for the sign (± 1)

$$\gamma_i := \text{sign}\frac{\partial F_i}{\partial u}(X, u).$$

When a derivative is identically zero, we define $\varphi_{ij} = 0$ or $\gamma_i = 0$.

A path π from the input u to a node X_j means, by definition, a sequence of k indices

$$\ell_1, \ell_2, \ldots, \ell_k = j$$

such that $\gamma_{\ell_1} \neq 0$ and

$$\varphi_{\ell_i, \ell_{i+1}} \neq 0$$

for all $i = 1, \ldots, k-1$. We denote by $s(\pi)$ the sign of the path, defined as the product

$$s(\pi) := \gamma_{\ell_1}\, \varphi_{\ell_1 \ell_2}\, \varphi_{\ell_2 \ell_3}\, \ldots\, \varphi_{\ell_{k-1} \ell_k}\,.$$

Similarly, a path from a node X_i to a node X_j means, by definition, a sequence of k indices

$$\ell_1, \ell_2, \ldots \ell_k = j$$

such that

$$\varphi_{i, \ell_1} \neq 0$$

and

$$\varphi_{\ell_i,\ell_{i+1}} \neq 0$$

for all $i = 1, \ldots, k-1$. We denote by $s(\pi)$ the sign of the path, defined as the product

$$s(\pi) := \varphi_{i\ell_1}\,\varphi_{\ell_1\ell_2}\,\varphi_{\ell_2\ell_3}\,\cdots\,\varphi_{\ell_{k-1}\ell_k}\,.$$

If there is a path from the input u to a node X_j, we say that X_j is *reachable*. If there is a path from a node X_i to the output node X_n, we say that the node X_i is *observable*.

We recall the statement of part 1 of the Theorem:

If the system is initially in steady state, the response of the output $x_n(t)$ will monotonically increase or decrease in time in response to changes in the input $u(t)$ if $u(t)$ is monotonically increasing or decreasing in time and all the directed paths from input node $u(t)$ to the output node $x_n(t)$ have the same parity. Furthermore, monotonically increasing (decreasing) $u(t)$ will trigger monotonic increase (respectively, decrease) of $x_n(t)$ if parity is positive or will trigger monotonic decrease (respectively, increase) if parity is negative.

Proof of part 1 of Theorem

We start by "pruning" those state variables X_j which do not lie in any path from the input node to the output node X_n. We now formalize this construction, which is analogous to the "Kalman decomposition" reduction to minimal systems in linear control theory [1]. We start by splitting the set of variables X into four disjoint subsets of variables $X = (x, y, z, w)$, as follows:

1. the output node X_n is a component of the vector x,
2. the components of x are reachable and observable,
3. the components of y are observable but not reachable,
4. the components of z are reachable but not observable, and
5. the components of w are neither reachable nor observable.

We assume without loss of generality that the output node X_n is in the first set of variables, x, since otherwise there would be no path from the input to output, and the output is then constant when starting a from steady state. It is clear that, with this partition, the equations look as follows:

$$\begin{aligned}
\dot{x} &= f(x, y, u) \\
\dot{y} &= g(y) \\
\dot{z} &= h(x, y, z, w, u) \\
\dot{w} &= k(y, w)
\end{aligned}$$

(for example, there cannot be a z nor w dependence in f and in g, since otherwise the z and/or w variables would be observable).

To prove part 1 of the Theorem, we need to show, for the original system $\dot{X} = F(X, u)$, that if we start from a steady state $F(X_0, u_0) = 0$ and if $u(t)$ is monotonic in time, with $u(0) = u_0$, then X_n will be also monotonic in time (with the same, or opposite, monotonic behavior depending on parity). Write $X_0 = (x_0, y_0, z_0, w_0)$, so $F(X_0, u_0) = 0$ means that

$$f(x_0, y_0, u_0) = g(y_0) = h(x_0, y_0, z_0, w_0, u_0) = k(y_0, w_0) = 0\,.$$

The assumption that all directed paths from the input node u to the output node X_n have the same parity applies also to the subsystem given by the variables in x in which the y variables are set to y_0:

$$\dot{x} = \hat{f}(x,u) = f(x,y_0,u) \tag{1}$$

with initial state $x(0) = x_0$, because partial derivatives of $\hat{f}$ with respect to x and u are also partial derivatives of the original F.

Suppose that we have already proved the theorem for this subsystem in which all variables are reachable and observable. We claim next that the same is then true for the original system. Consider the solution $x(t)$ of (1) with input $u = u(t)$ and $x(0) = x_0$. Consider also the solution of the full system $\dot{X} = F(X,u)$ with $X(0) = X_0$ and the same input u, and write it in the corresponding block form

$$X(t) = (\xi(t), \psi(t), \zeta(t), \omega(t)).$$

We want to prove that $\xi(t) = x(t)$ for all $t \geq 0$, from which the claim will follow. But this just follows because $g(y_0) = 0$ implies that $y(t) \equiv y_0$. (Note that the variables $\zeta(t)$ and $\omega(t)$ do not affect the output variable, which is a component of $\xi(t)$.)

We now prove the theorem for the x-subsystem, for which all variables are reachable and observable. For ease of notation, we will write $\hat{f}$ simply as f, use n for the size of x, and assume that the output node is x_n. Pick any index $i \in \{1,\ldots,n\}$. By reachability, there is at least one path π from the input to x_i and, if $i < n$, then by observability there is at least one path θ from x_i to the output node x_n. We claim that every other path π' from the input to x_i has the same parity as π. Suppose without loss of generality that the parity of π is $+1$. We need to see that every other path π' from the input to x_i also has parity $+1$. If $i = n$, this is true by assumption (all paths from input to output have the same parity). So assume $i < n$. Suppose that π' has parity -1. Then, the path $\pi\theta$ obtained by first following π and then following θ has parity $(+1) * \rho = \rho$, where ρ is the parity of θ, and the path $\pi'\theta$ obtained by first following π' and then following θ has parity $(-1) * \rho = -\rho$. So we have two paths from input to output with different parity, which contradicts the assumption of the Theorem. In conclusion, every two paths from the input to any given node have the same parity.

We assign a label with values "$+1$ or -1" σ_u and σ_i, $i = 1,\ldots,n$, to the nodes u and each node $x_1,\ldots,x_n$ respectively, as follows: $\sigma_u := +1$, $\sigma_i :=$ sign of any path from u to x_i. A key observation is that, if $\varphi_{ij} = +1$ then $\sigma_i = \sigma_j$, and if $\gamma_i = +1$ then $\sigma_u = \sigma_i$. Indeed, if we have a path π from the input to x_i, then a path π' can be obtained, from the input to x_j, by simply adjoining the edge from i to j, which has parity equal to the parity of π. Since σ_j is the sign of any path from the input to x_j, it follows that $\sigma_i = \sigma_j$, as claimed. The statement for $\gamma_i = +1$ is simply (since we defined $\sigma_u := +1$) that $\sigma_i = +1$ if the one-step path from the input to node x_i has parity 1, which means that all paths have this parity. Similarly, if $\varphi_{ij} = -1$ then $\sigma_i = -\sigma_j$, and if $\gamma_i = -1$ then $\sigma_u = -\sigma_i$.

Now make the change of variables $x_i \mapsto \sigma_i x_i$ (i.e., reverse the sign of variables with a "-1" label). Writing the system in the new variables, we have now that

$$\frac{\partial f_i}{\partial u}(x,u) \geq 0 \tag{2}$$

for all $i = 1,\ldots,n$ and

$$\frac{\partial f_j}{\partial x_i}(x,u) \geq 0 \tag{3}$$

for all $i,j = 1,\ldots,n$. Thus in the new variables we have what is called a *cooperative system* [2].

We must prove that, if $u = u(t)$ is a monotonically increasing input for a cooperative system, and if $x(0) = x_0$ is a steady state $f(x_0,u_0) = 0$, then every coordinate $x_i(t)$ of $x(t)$ (and, in particular, the output node)

is monotonically increasing as well. (In the original coordinates, before sign reversals, $x_i(t)$ will decrease if $\sigma_i = -1$.) Similarly if $u = u(t)$ is a monotonically decreasing input for a cooperative system, and if $x(0) = x_0$ is a steady state $f(x_0, u_0) = 0$, then every coordinate $x_i(t)$ of $x(t)$ (and, in particular, the output node) is monotonically decreasing as well. We prove the increasing statement, since the second statement is proved analogously. From now on, for any two vectors $a, b \in \mathbb{R}^n$, we write simply $a \leq b$ to mean that $a_i \leq b_i$ for each $i = 1, \dots, n$.

We let $\varphi(t, x_0, v)$ denote the solution of $\dot{x} = f(x, u)$ at time $t > 0$ with initial condition $x(0) = x_0$ and input signal $v = v(t)$. *Kamke's Comparison Theorem* (see [2] for systems without inputs, and [3] for an extension to systems with inputs), asserts as follows: Let $y(t)$ and $z(t)$ be two solutions of the system $\dot{x} = f(x, u)$ corresponding, respectively, to an input $v(t)$ and an input $w(t)$. Suppose that $y(0) \leq z(0)$ and that $v(t) \leq w(t)$ for all $t \geq 0$. Then, $y(t) \leq z(t)$ for all $t \geq 0$.

Now pick an input v that is non-decreasing in time and an initial state x_0 that is a steady state with respect to $v_0 = v(0)$, that is, $f(x_0, v_0) = 0$. Since $v(t)$ is non-decreasing, we have that $v(t) \geq v(0)$ so that, by comparison with the input that is identically equal to $v(0)$, we know that

$$\varphi(h, x_0, v) \geq \varphi(h, x_0, v_0)$$

for all $h \geq 0$, where, by a slight abuse of notation, "v_0" is the function that has the constant value v_0. We used the comparison theorem with respect to inputs and with the same initial state. The assumption that the system starts at a steady state gives that $\varphi(h, x_0, v_0) = x_0$ for all $h \geq 0$. Therefore:

$$x(h) \;\geq\; x(0) \qquad \text{for all } h \geq 0\,. \tag{4}$$

Next, we consider any two times $t \leq t+h$. We wish to show that $x(t) \leq x(t+h)$. Using (4) and the comparison theorem now applied with respect to initial states and the same input, we have that:

$$x(t+h) \;=\; \varphi(t, x(h), v_h) \;\geq\; \varphi(t, x(0), v_h)\,,$$

where v_h is the "tail" of v, defined by: $v_h(s) = v(s+h)$. On the other hand, since the function v is non-decreasing, it holds that $v_h \leq v$, in the sense that the inputs are ordered: $v_h(t) \leq v(t)$ for all $t \geq 0$. Therefore, using once again the comparison theorem with respect to inputs and with the same initial state, we have that

$$\varphi(t, x(0), v_h) \;\geq\; \varphi(t, x(0), v) = x(t)$$

and thus we proved that $x(t+h) \geq x(t)$. So x is a non-decreasing function. This concludes the proof. See [4] for a related result.

We recall the statement of part 2 of the Theorem:

If the system is initially in steady state, the response of the output $x_n(t)$ will monotonically increase or decrease in time in response to changes in the input $u(t)$ if all the directed paths from the input nodes to the output node pass through an internal node $x_i(t)$ with monotonically increasing or decreasing dynamics and all the directed paths from input node $x_i(t)$ to the output node $x_n(t)$ have the same parity. Furthermore, monotonically increasing (decreasing) $x_i(t)$ will trigger monotonic increase (respectively, decrease) of $x_n(t)$ if parity is positive or will trigger monotonic decrease (respectively, increase) if parity is negative.

Proof of part 2 of Theorem

The assumption that all directed paths from the input node u to the output node X_n must pass through the internal node X_i can be formalized by splitting the set of nodes X into three subsets, $X = (x, y, z, w)$, where

1. the components of x are those nodes X_j, $j \neq i$, for which there is at least one path from the input node u to X_j which does not pass through node X_i,

2. $y = X_i$, and

3. the components of z are all remaining nodes, including X_n.

For this partition, the equations look as follows:

$$\begin{aligned} \dot{x} &= f(x, y, z, u) \\ \dot{y} &= g(x, y, z, u) \\ \dot{z} &= h(z, y) \end{aligned}$$

because, if there were any dependence of h on some coordinate x_j, then there would be a path from the input to some component of z (follow a path to x_j and concatenate it with an edge from x_j to this component).

The condition that all the directed paths from $y = X_i$ to the output node X_n have the same parity means that in the system

$$\dot{z} = h(z, v)$$

(where we now view $y(t)$ as an input, which we write as "$v(t)$" to avoid confusion) all paths from the input to the output have the same parity, as in the hypothesis of part 1 of the Theorem. Suppose that we consider an input u, starting from a steady state (x_0, y_0, z_0). Think of $v(t) = y(t)$ as an input. Since we started from a steady state, we know that $h(v(0), z_0) = 0$. This, if $v(t)$ is monotonic, part 1 of the theorem gives us that the output is monotonic, increasing or decreasing depending on parity and the increasing or decreasing character of the input. So part 2 is proved as well.

2 A result on steady-state gains

Suppose that now that

$$\dot{X} = F(X, u)$$

is a system with the following two properties:

1. for each nondecreasing input $u(t)$, and any trajectory starting from a steady state $F(X_0, u(0)) = 0$, the output $X_n(t)$ is nondecreasing, and

2. for each constant input $u(t) \equiv \bar{u}$, and any initial state, the output $X_n(t)$ converges to a value $G(\bar{u})$ that depends only on $\bar{u}$ (and not on the initial state, which need not be a steady state).

Observe that the first condition holds provided that all the directed paths from input node $u(t)$ to the output node $X_n(t)$ have positive parity. (An analogous result to the one stated below holds if all paths have negative parity and the output is nonincreasing.)

We call G the *i/o steady state response* of the system. Such a function exists, for example, if for each constant input $\bar{u}$ there is a (necessarily unique) globally asymptotically stable steady state of $\dot{X} = F(X, \bar{u})$.

Proposition. For each nondecreasing input $u(t)$, and each $T \geq 0$, $X_n(T) \leq G(u(T))$.

Proof. Consider a nondecreasing input $u(t)$ and a steady state $F(X_0, u(0))$. Fix an arbitrary time $T \geq 0$. Let $v(t)$ be the nondecreasing input that is defined by $v(t) := u(t)$ if $t \leq T$ and $v(t) = u(T)$ for all $t > T$. Then the output $X_n(t)$ corresponding to solving $\dot{X} = F(X, v(t))$ with $X(0) = x_0$ is nondecreasing, by assumption,

and so in particular $X_n(T) \leq X_n(t)$ for all $t > T$. Now consider the initial state $X(T)$ and the constant input $w(t) \equiv u(T)$. It follows from the second assumption that $X_n(t) \to G(u(T))$ as $t \to +\infty$, which together with $X_n(T) \leq X_n(t)$ for all $t > T$ gives that $X_n(T) \leq G(u(T))$, as desired.

References

[1] E.D. Sontag. *Mathematical Control Theory. Deterministic Finite-Dimensional Systems*, volume 6 of *Texts in Applied Mathematics*. Springer-Verlag, New York, second edition, 1998.

[2] H. Smith. *Monotone Dynamical Systems: An Introduction to the Theory of Competitive and Cooperative Systems, Mathematical Surveys and Monographs, vol. 41*. AMS, Providence, RI, 1995.

[3] D. Angeli and E.D. Sontag. Monotone control systems. *IEEE Trans. Automat. Control*, 48(10):1684–1698, 2003.

[4] D. Angeli and E.D. Sontag. Remarks on the invalidation of biological models using monotone systems theory. In *Proc. IEEE Conf. Decision and Control, Maui, Dec. 2012*, 2012. Paper TuC09.3.
